# Inferring transcriptional compensation interactions in yeast via stepwise structure equation modeling

**DOI:** 10.1186/1471-2105-9-134

**Published:** 2008-03-03

**Authors:** Grace S Shieh, Chung-Ming Chen, Ching-Yun Yu, Juiling Huang, Woei-Fuh Wang, Yi-Chen Lo

**Affiliations:** 1 Institute of Statistical Science, Academia Sinica, Taipei, 115, Taiwan; 2 Institute of Biomedical Engineering, National Taiwan University, Taipei, 106, Taiwan; 3 Institute of Cellular and Organismic Biology, Academia Sinica, Taipei, 115, Taiwan

## Abstract

**Background:**

With the abundant information produced by microarray technology, various approaches have been proposed to infer transcriptional regulatory networks. However, few approaches have studied subtle and indirect interaction such as genetic compensation, the existence of which is widely recognized although its mechanism has yet to be clarified. Furthermore, when inferring gene networks most models include only observed variables whereas latent factors, such as proteins and mRNA degradation that are not measured by microarrays, do participate in networks in reality.

**Results:**

Motivated by inferring transcriptional compensation (TC) interactions in yeast, a stepwise structural equation modeling algorithm (SSEM) is developed. In addition to observed variables, SSEM also incorporates hidden variables to capture interactions (or regulations) from latent factors. Simulated gene networks are used to determine with which of six possible model selection criteria (MSC) SSEM works best. SSEM with Bayesian information criterion (BIC) results in the highest true positive rates, the largest percentage of correctly predicted interactions from all existing interactions, and the highest true negative (non-existing interactions) rates. Next, we apply SSEM using real microarray data to infer TC interactions among (1) small groups of genes that are synthetic sick or lethal (SSL) to SGS1, and (2) a group of SSL pairs of 51 yeast genes involved in DNA synthesis and repair that are of interest. For (1), SSEM with BIC is shown to outperform three Bayesian network algorithms and a multivariate autoregressive model, checked against the results of qRT-PCR experiments. The predictions for (2) are shown to coincide with several known pathways of Sgs1 and its partners that are involved in DNA replication, recombination and repair. In addition, experimentally testable interactions of Rad27 are predicted.

**Conclusion:**

SSEM is a useful tool for inferring genetic networks, and the results reinforce the possibility of predicting pathways of protein complexes via genetic interactions.

## Background

While the existence of genetic compensation is widely accepted, the mechanism is largely unknown but important [1,2]. The proposed algorithm (SSEM) was motivated by inferring transcriptional compensation (TC) networks of SGS1 (or RAD27) and its synthetic sick or lethal (SSL) partners [3,4]. However, SSEM can also be applied to infer other types of networks, such as transcriptional regulatory networks. Following a gene's loss, the expression level of its compensatory gene increases (decreases), this phenomenon is called TC (transcriptional diminishment, abbreviated as TD). Paralogs or redundant genes are called digenic SSL gene pairs if the combination of two mutants, neither by itself lethal, causes the organism to die or malfunction [3,5,6]. SSL effects underlie many complex human diseases, such as type II diabetes, schizophrenia, Alzheimer's disease, and others [4]. Since genetic networks derived from model organisms, such as yeast, are likely to be conserved in humans the prediction of TC and TD may shed light on pathways that cause complex human diseases. With the abundant information produced by microarray technology, various approaches have been proposed to infer genetic networks or transcriptional regulatory networks. Most of them may be classified into three classes, namely, graph models, discrete variable models and continuous variable models. Due to space limits, we refer to [7] (in Additional file 1) for a thorough review of the models.

Graph models (for instance, [8]) depict genetic interactions through directed graphs or digraphs instead of characterizing the interactions quantitatively. Some graph models simply reveal structural information, others annotate the directions and signs of the regulations among genes. Because of their simplicity, graph models usually require much less data than models in the other two categories. But they are inherently static and may not capture the dynamics of genetic regulations and the simultaneous regulation of a given gene by multiple genes. Discrete variable models discretize gene expressions into a few states. The dynamics of gene expressions may be perceived as transitions of finite states. Typical discrete variable models proposed are Boolean networks, probabilistic Boolean networks and discrete Bayesian networks (for details, see a classic paper [9]). Continuous variable models characterize the expression of a gene or its change by a linear or non-linear continuous function of the expression of other genes. The genetic interactions are frequently modeled by a first-order or a second-order differential (or difference) equation. Continuous variable models consist of two major types: continuous Bayesian networks [10-12] and deterministic differential systems [13].

Although each class of models has been shown to be informative for understanding genetic interactions, most of the models, except some Bayesian networks, have the estimation bias problem due to model mis-specification. The model mis-specification arises from the fact that microarrays measure the mRNA expressions only, while genetic interactions may be influenced by enzymes or proteins, for instance transcriptional factors. Furthermore, most genetic networks reconstructed in previous studies considered only a subset of the whole genome. Consequently, those genes that were left out may be regarded as latent factors influencing the genes of interest. Thus, ignoring latent factors in the models may cause bias when inferring the genetic interactions. Although a Bayesian network can also incorporate latent factors [11,12], the amount of data required may prevent it from being used.

To account for the latent factors effect using a reasonable amount of microarray data, a stepwise structural equation modeling algorithm (SSEM) is proposed in this article. SSEM is based on structural equation modeling (SEM) [14], which unifies factor analysis and path analysis. Assuming linear relations among the observed and latent variables, the basic idea of SEM is to minimize the discrepancy between the fitted covariance matrix and the sample covariance matrix. Zhou *et al*. [15] used shortest path analysis to identify transitive genes between two given genes in the same biological pathway. Xie and Bentler [16] showed that the latent factors can be identified and their relations may be estimated reasonably by SEM. Note that without identifying the latent factors reasonably, the causal relations among genes can not be estimated correctly.

In this article, we extend the model in [16] to simultaneously infer both latent factor-gene and gene-gene interactions. Both [16] and SSEM extend the methodology of SEM in the sense that the latent factors are formed from data and not chosen a priori from domain knowledge as commonly practiced in the social sciences. SSEM learns genetic interactions by both exploratory factor analysis (EFA) and SEM with various model selection criteria (MSC) in a stepwise fashion. The incorporation of MSC helps SSEM circumvent the overfitting problem. The performance of SSEM with six different MSC is evaluated using two sets of simulated networks to determine which MSC works best. The software SSEM automatically runs through all of the steps of SSEM, and outputs predicted gene interactions. Finally, SSEM is applied to infer TC and TD interactions for (1) small groups of genes that are synthetic sick or lethal (SSL) to SGS1, and (2) SGS1 or RAD27 with their SSL partners from 51 genes involved in yeast DNA synthesis and repair that are of interest. Both predictions are verified by an extensive quantitative RT-polymerase chain reaction experiment (qRT-PCR); see Additional file 2 for details.

## Results and discussion

The MSC suitable for predicting genetic networks remains unknown, while an adequate MSC can prevent the algorithm from overfitting. Thus we have carried out an extensive simulation to evaluate eight criteria used in commercial SEM software, such as Mplus version 3 [17]. The results of the top six MSC *χ*^2^/*df, χ*^2^-*df*, Mean square error (MSE), Akaike information criterion (AIC), Bayesian information criterion (BIC), and adjBIC are reported in Additional file 3. SSEM with BIC outperforms all of the others. Since the network topology, latent factors (**x**(*t*)), gene-gene interactions (**w**), and latent factor-gene regulations (**Λ**) are well defined for the simulated data, exact quantitative performance can be computed.

### Results of SSEM with various MSC using simulated data

Time course data from 6-gene and 10-gene regulatory networks with two latent factors are generated. The simulation consists of various sample sizes and noise levels. Let *x*_*i*_(*t*), *y*_*i*_(*t*) and *ε*_*i*_(*t*) denote the expression level of latent factor *i*, gene *i *and noise variable *i*, respectively. The linear dynamic factor model (LDFM, see Section 4.1 for general model setting) to generate the 6-gene network is as follows:

(1) $$\begin{array}{l} y_{1}(t)=0.5x_{1}(t)+0.5y_{1}(t-1)+0.6y_{2}(t-1)+\varepsilon_{1}(t)\\ y_{2}(t)=0.7x_{1}(t)+0.5y_{2}(t-1)+0.4y_{3}(t-1)+\varepsilon_{2}(t)\\ y_{3}(t)=0.7x_{1}(t)+0.5y_{3}(t-1)+0.4y_{4}(t-1)+0.5y_{5}(t-1)+\varepsilon_{3}(t)\\ y_{4}(t)=0.6x_{2}(t)+0.6y_{5}(t-1)+\varepsilon_{4}(t)\\ y_{5}(t)=0.7x_{2}(t)+0.5y_{5}(t-1)+\varepsilon_{5}(t)\\ y_{6}(t)=0.5x_{2}(t)+0.5y_{4}(t-1)+0.4y_{6}(t-1)+\varepsilon_{6}(t) \end{array}$$

where *x*_1_(*t*) ~ *N*(0,0.1), *x*_2_(*t*) ~ *N*(0,0.1), *y*_*i*_(0) ~ *U*(0,1), and *ε*_*i*_(*t*) ~ *N *(0, $\sigma_{i}^{2}$), *i *= 1,...,6. Note that $\sigma_{i}^{2}$ is determined by the variance of *y*_*i*_(*t*) and a pre-specified noise level. The noise level is quantified by a contrast-to-noise ratio (CNR), defined as the ratio of the signal standard deviation to the noise standard deviation. *CNR *= 1.3 or 2.0 corresponds to high or medium noise levels, respectively. For the 10-gene network, we refer to Equation (5) of Simulation.pdf of the Supplementary data.

Note that both the 6- and 10-gene networks are sparse, which roughly follow the sparse property of *cis*-regulatory networks [18]. For each network, time course data are simulated under various conditions; sample sizes (*T =T*_min_,*50 *or *100*) and noise levels, where *T *is the number of time points and *T*_min _= 2*n *+ 1. Without incorporating any biological knowledge, for a fully connected n-gene network (namely all interactions are non-vanished), *T *= 2n+1 is the minimum number of time points required (denoted as *T*_min_) for proper estimation of $\hat{\Sigma }$ in (4), and hence for all parameters in the model. However, the latest version of SSEM can be iterated from a non-fully connected network, and hence the restriction *T *= 2n+1 no longer exists. Table 1 summarizes the performance of SSEM with AIC and BIC for the 6-gene network under various settings of (*CNR*, *T*). The averages of the true positive rate (TPR), true negative rate (TNR), and false positive rate (FPR) for the top 1 (top 5) networks, in terms of MSC value, in 100 experiments are reported. TPR (also known as sensitivity) is the percentage of correctly predicted links from the total existing links (positives) in the simulated network. Likewise, TNR (specificity) is the percentage of correctly predicted non-existing links (negatives) out of the total non-existing links in the simulated network. Clearly, SSEM with BIC outperforms SSEM with AIC, and the results from the 10-gene network also confirm this.

**Table 1:** Performance of SSEM with BIC and AIC applied to the 6-gene network with various combinations of (CNR, T).

		Top 1 model			Top 5 models		
		
(*CNR, T*)	MSC	TPR (%)	TNR (%)	FPR (%)	TPR (%)	TNR (%)	FPR (%)
(2.0, 100)	BIC	97.3	97.1	2.9	97.0	95.3	4.7
	AIC	97.7	85.1	14.9	97.7	84.4	15.6
(2.0, 50)	BIC	84.6	87.7	12.3	83.9	86.2	12.9
	AIC	88.7	78.9	21.1	88.0	78.0	21.1
(2.0, 13)	BIC	80.9	79.1	20.9	71.8	63.5	36.5
	AIC	81.8	80.4	19.6	72.1	64.0	36.0
(1.3, 100)	BIC	84.9	90.0	10.0	84.5	88.6	11.4
	AIC	88.7	78.8	21.2	88.8	78.5	21.5
(1.3, 50)	BIC	73.5	83.5	16.5	72.5	82.4	16.0
	AIC	78.9	72.7	27.3	78.1	71.8	27.0
(1.3, 13)	BIC	77.1	75.4	24.6	68.9	61.0	39.0
	AIC	78.0	76.7	23.3	69.5	61.5	38.5

We further compared SSEM with BIC to VBEM [12] using the 6- and 10-gene networks in Simulation.pdf. The results are in Table 2, and SSEM with BIC outperforms VBEM in terms of TPRs for both networks; details are provided in Simulation.pdf.

**Table 2:** Performance of SSEM with BIC and VBEM applied to the 6-gene and 10-gene networks with various combinations of (n, CNR, T)

	SSEM with BIC			VBEM		
	
(n,*CNR, T*)	TPR (%)	TNR (%)	FPR (%)	TPR (%)	TNR (%)	FPR (%)
(6,2.0, 13)	81.8	80.4	19.6	0.0	96.0	4.0
(6,1.3, 13)	78.0	76.7	23.3	0.0	100.0	0.0
(10,2.0, 21)	62.0	67.0	33.0	6.0	94.0	6.0
(10,1.3, 21)	60.0	62.0	38.0	11.0	90.0	10.0

### Results on real time course microarray data

In this section, SSEM is first applied to infer TC/TD interactions for small groups of genes SSL to SGS1, for example CSM3, MUS81, SIS2, SWE1and TOP1 in [3]. Next, SSEM infers TC/TD interactions from SGS1 or RAD27 SSL gene pairs, formed from 51 genes involved in yeast DNA synthesis and repair. SGS1 encodes a RecQ DNA helicase, of which the homologues in human cells include the WRN, BLM and RECQ4 genes. Mutation of the SGS1 gene results in premature aging in yeast mother cells as well as genome instability. Further, these genes and their processes are highly conserved in eukaryotic cells, and mutations in these genes may lead to cancer-predisposition syndromes and symptoms resembling premature aging [4]. On the other hand, Rad27 encodes a structure-specific (5'-flap) endonuclease which has a human homolog, FEN1; Rad27 has a distinct role in processing Okazaki fragments during DNA synthesis in the S phase. Deletion of RAD27 in cells also causes hypersensitivities to various DNA damaging agents [19]. Rad27 was shown to be necessary for maintaining genome stability by restricting DNA recombination between short repeated sequences and processing long-patch base excision repair [20-23].

cDNA microarray data from the *alpha*, *cdc15 *and *cdc28 *experiments in [24] were applied to the four algorithms to infer the gene network of interest. The *elu *data set was not included because it was synchronized differently from *alpha*, *cdc15 *and *cdc28*. The experiment and control groups were mRNAs extracted from synchronized and non-synchronized yeast cultures, respectively. The synchronization was conducted by treating yeast cultures with alpha factor arrest and arrests of a temperature-sensitive mutant *cdc15 *and mutant *cdc28*. A full description and complete data sets are available at [25]. The red (R) and green (G) fluorescence intensities were measured from the mRNA abundance in the experiment group and control group, respectively. There were 18, 24 and 17 time points in the *alpha*, *cdc15 *and *cdc28 *data sets with no replicates; we first aggregated these three datasets to increase the number of time points to 59. This aggregation was applied in [16], and it resulted in some meaningful gene networks.

Log ratios of the six genes' expression levels were fitted to SSEM with BIC, VBEM [12], MAPEM [26] and LDS [11] algorithms. The results were checked against qRT-PCR results (see Figure 1 in Additional file 4). Excluding latent factor-gene interactions, the modified true positive rate (mTPR) of the top model selected by SSEM with BIC equals 7/12. While the mTPRs of VBEM, MAPEM and LDS equal 2/12 (at 99% significance level), 6/12 and 0, respectively. Fitting five genes' expression to a multivariate AR(1) model resulted in 0/12 mTPR; see Additional file 5 for details. This shows how latent factors improve the estimation of gene interactions $\hat{W}$ and thus mediate proper extraction of biological knowledge. We also ran SSEM when the sample size was small (T = 11) for the 6-gene network, and the mTPR of the top model predicted by SSEM equaled 7/12. For this application, SSEM took about 19 minutes on PCs with Pentium IV 3.4 GHz and 2.5 GB RAM.

Next, SSEM was applied to infer TC/TD interactions among the 51 genes that are SSL to SGS1 or RAD27. Our collaborator has conducted extensive qRT-PCR experiments (in Additional file 6) to verify that among these predictions, SSEM successfully uncovered several TC/TD interactions of SGS1 with genes involved in DNA replication (e.g., SRS2, PLO32, RNR1, SLX1, MUS81 and TOP1), DNA repair (e.g., RAD51 and RAD52), checkpoint arrest (RAD9) and chromosome segregation (CSM3). These genetic interactions are consistent with the following experimental results from published literature. Sgs1 and Srs2 are known redundant pathways in replication [27,28]; for instance, Srs2 and Sgs1-Top3 suppress crossovers during double strand break repair in yeast. Further, defects in RAD51 and other homologous recombination genes suppressed synthetic lethality/sickness of the double mutant *sgs1Δ srs2Δ*. Slx1-Slx4 was found to be a second structure specific endonuclease functionally redundant with Sgs1-Top3 in [29]. The Sgs1/Top3/Rmi1 and Mus81/Mms4 complexes are involved in both double-strand break repair and homologous recombination [30]. This indicates that Sgs1/Top3/Rmi1 and Mus81/Mms4 are alternative pathways to resolve recombination intermediates. [31] identified that Sgs1 participated in a RAD52-dependent recombination pathway. [32] found that Rad9 and Sgs1 interacted genetically and possibly physically. Cells lacking Sgs1 frequently arrest as large-budded cells with a single nucleus in the mother cell, or "stuck" between mother and daughter cells, which resulted in missegregation during mitosis [33,34], whereas Csm3 is required for DNA replication checkpoint and accurate chromosome segregation. Similarly, SSEM was applied to predict the interactions of the fifteen SSL pairs of RAD27, and among them HPR5, SGS1, MUS81, ESC2, HST1, HST3 and CSM3 had TC interactions with RAD27, whereas RAD52, HPR5, SIS2, SOD2, HPC2, LYS7, RAD9, RAD51 and RAD54 had TD interactions with RAD27. For the second application, SSEM took about 3 to 4 hours on PCs with Pentium IV 3.0 GHz and 1 GB RAM.

The results involving SGS1 reinforce the possibility of applying genetic interactions to predict pathways of protein complexes [35]. The predictions of RAD27 are intriguing to biologists since biological experiments to screen all possible interactions have been prohibitive thus far. Note that SSEM can also be applied to infer TC interactions of 872 SSL gene pairs in [3,4] or other large networks with a similar structure, for instance the other six groups of SSL pairs involving ARC40, ARP2, BBC1, BIM1, BNI1 and NBP2. The large network of 887 SSL pairs can be broken down to subgroups that center on SGS1, RAD27, the above six genes, and other hub genes. Then each subgroup can be inferred individually, similarly to the group involving SGS1.

## Conclusion

The novelties and merits of SSEM are as follows. First, SSEM expands the scope of application of most algorithms in the area of gene networks. Specifically, SSEM is shown to predict several TC/TD interactions of SGS1 accurately, verified by qRT-PCR experiments, and these interactions coincide with existing pathways. Further, SSEM predicts a few novel TC/TD interactions involving RAD27, and these predictions can be verified by biological experiments. Importantly, SSEM can be further applied to predict genetic interactions of other large networks with a similar structure, while biological experiments to screen all possible interactions may be prohibitive. Second, SSEM extends the approach in [16] such that it can infer both latent factor-gene and gene-gene interactions simultaneously. Third, SSEM incorporates an MSC in a stepwise fashion to circumvent the overfitting problem. Although SSEM was shown to infer genetic networks using time course data with no replicates, it can also be applied to short time course data with replicates by modifying the terms involved in replicates and the sample size. As technology advances, we anticipate more data sets with replicates to become available and a greater demand for algorithms like SSEM to infer gene networks.

Using SSEM with the model in Equation (2) has been shown to outperform fitting a multivariate autoregressive model straightforwardly. This demonstrates the important role of latent factors and the efficiency of SSEM. Further, SSEM outperforms three Bayesian network algorithms that impose linear models on latent factors, while SSEM does not assume any structure on latent factors. However, SSEM shares one drawback with continuous Bayesian networks. Both approaches assume that the vector of log ratios of gene expression **y**(*t*) follows a multivariate normal distribution. This assumption may limit its application, although log ratios of gene expression do follow a normal distribution in most cases.

Although SSEM may serve as an exploratory tool for genetic interactions, the model in (2) is an approximation to the true model, and BIC is a large-sample result. Further improvements for future research include finding a novel MSC for SSEM when the sample size is small, and developing a nonlinear model with latent factors or a lag-k and *k *> 1 in time to model genetic interactions. The goal of SSEM is to model small to medium networks with precise prediction instead of modeling large or genome-wide networks with inaccurate prediction. Some results on incorporating various types of data, e.g. motif information, and ChIP-chip data besides microarray data, to predict transcriptional modules have been explored in the literature [36-38]. However, integrating various types of data for reliable prediction of complex genetic networks remains a challenging topic, and we leave this for future research.

## Methods

### The linear dynamic factor model

We assume that time course microarray data follow an LDFM, which includes both factor-gene and gene-gene regulation in the model. Let ${\tilde{y}}_{i}(t)$ denote the expression of gene *i *at time *t *for 1 ≤ *i *≤ *n*, where *n *is the number of genes in the network. Further, let *y*_*i*_(*t*) be the centered ${\tilde{y}}_{i}(t)$, namely $y_{i}(t)={\tilde{y}}_{i}(t)-{\bar{\tilde{y}}}_{i}$, where ${\bar{\tilde{y}}}_{i}$ is the mean of ${\tilde{y}}_{i}(t)$ over time. We incorporate centered variables to avoid an intercept term in Equation (2) to reduce *n *parameters that are not of interest. Specifically, LDFM assumes that *y*_*i*_(*t*) is regulated by a linear combination of latent factors at time *t *and centered observed variables (genes) at time (*t *- 1), and the regulation is invariant across time as follows.

(2) **y**(*t*) = **Λx**(*t*) + **Wy**(*t *- 1) + **ε**(*t*),

where **y**(*t*) is the vector of the expression levels of the *n *genes at time *t*, **x**(*t*) is the (*k *× 1) vector of the latent factors' expression at time *t*, and *ε*(*t*) is the (*n *× 1) noise vector that assumes *N*_*n *_($\vec{0}$, *Q*), where *Q *is a diagonal covariance matrix. Further, **Λ **is the (*n *× *k*) latent interaction matrix, in which *λ*_*ij *_denotes the influence of latent factor *j *on gene *i *at the same time, and **w **is the gene-gene interaction matrix, in which *W*_*ij *_denotes the influence of gene *j *at time (*t *- 1) on gene *i'*s expression at time *t*. Latent factors **x**(*t*) are assumed to follow *N*_*k *_($\vec{0}$, Σ_*k*_), and **x**(*t*) and **ε**(*t*) are uncorrelated such that the model is identifiable. Applying biological knowledge, SSEM can infer sufficiently large networks. For example, when inferring TC interactions from SSL pairs, interactions (*W*_*ij*_'s) are non-vanished only for SSL pairs. For instance, when predicting TC interactions of SSL gene pairs involving SGS1, fitting one equation $SGS1(t)=\Sigma_{i=1}^{k}\lambda_{i}F_{i}+\Sigma_{i=1}^{23}W_{i}y_{i}(t-1)$ is sufficient, where *y*_*i*_*'s *are the twenty-three genes that are SSL to SGS1 [3,4], and the other *W*_*j*_'s are vanished for gene *j *that is not SSL to SGS1. The aforementioned equation can be inputed into the latest version of SSEM as an initial network, and when no links are specified to be deleted in the iteration, SSEM will predict gene-gene interactions for the non-vanished *W*_*j*_'s, and infer the factor-gene interactions from data. Note that when inferring transcriptional regulatory networks, Equation (2) is also able to model the combination of multiple genes to activate (or repress) a target gene simultaneously. The major difference between LDFM and the state space model (SSM), for example the model in [12], is that the former does not model interactions among latent factors across time.

SEM is adopted since it considers latent factor-gene and gene-gene interactions simultaneously to reveal gene networks using microarray data. An SSEM algorithm is introduced to learn the parameters **Λ **and W in LDFM. The main idea is to learn the regulation network iteratively. In each iteration, for a generated network, we estimate the parameters by SEM and evaluate its goodness-of-fit. The top few networks of each iteration are retained for the next iteration, until the optimal network, in terms of any MSC, emerges. SSEM is available to users upon request from the corresponding author.

SSEM consists of three parts. Specifically, in Part 1, EFA is applied to learn some initial latent structures, which specify latent factor-gene interaction. In Part 2, networks consist of any given initial latent structure and (randomly generated) partially connected gene-gene interactions are considered. SEM is applied to estimate **x**(*t*), **Λ **and **w **of any network considered, and a specified MSC evaluates the goodness-of-fit of the network. In Part 3, plausible networks are generated by systematically and iteratively eliminating insignificant links (interactions) based on the associated *t*-statistics resulting from SEM. These three parts are described in detail in the learning networks section.

### Learning the initial latent structures

Incorporating a correct latent structure is crucial for reconstructing genetic networks. First, EFA is employed to learn potential latent structures to start the iterative process. EFA is a common practice to ascertain the latent factors that influence the observed variables. Fundamentally, factor analysis assumes that there are some latent factors, fewer in number than genes, that are responsible for the co-variation among the observed gene expressions. EFA may be expressed as

(3) $$y(t)=\tilde{\Lambda }\tilde{x}(t)+\tilde{u}(t),$$

where $\tilde{x}(t)$, $\tilde{\Lambda }$ and $\tilde{u}(t)$ are all estimated without taking gene-gene interaction into account. Specifically, $\tilde{x}(t)$ is (*m *× 1) the common factors at time *t*, $\tilde{\Lambda }$ is the *n *× *m *latent interaction matrix, in which ${\tilde{\lambda }}_{ij}$ denotes the influence of latent factor *j *on the expression of gene *i *at time *t *estimated without explicitly taking account of gene-gene interaction, and $\tilde{u}(t)$ is (*m *× 1) the unique factors at time *t *that can not be explained by the common factors $\tilde{x}(t)$. Comparing Equations (2) and (3), the latent structure embedded in $\tilde{\Lambda }\tilde{x}(t)$ would deviate from the true one except when the factor $\tilde{u}(t)$ accounts for the effect of gene-gene interaction, that is, equal to **w**_**y**_(*t *- 1). This shows that fitting a structural equation model with the latent factors estimated solely by EFA to the gene expressions [16] may not result in the correct latent structure. Therefore, given *k *latent factors suggested by EFA, we consider three possible numbers of latent factors (*k*-1, *k *or *k*+1), along with the associated latent structure in Part 1 of SSEM. The common factors are extracted by a principal component analysis with promax oblique rotation, which rotates factors in order to fit a hypothesized structure of latent factors.

Determining the number of common factors that best explain the observed variables is one of the practical issues in EFA. Various guidelines have been proposed, for instance, eigenvalue ≥ 1 [39] and the scree test [40]. Different guidelines may lead to different choices. Based on the "weaker lower bound" suggested by [40], SSEM searches through *k *- 1, *k*, and *k *+ 1 common factors and the associated latent structures, where *k *is the number of common factors with eigenvalues ≥ 1 resulting from EFA. Then, for each given *k*, the latent structure is obtained by eliminating the links with factor loading less than a constant, which can be specified by users and the default value is 0.2.

### Network (model) selection criterion

In the iterations of SSEM, latent factor **x**(*t*), and the parameters **Λ **and **w **of a given network are estimated, and the goodness-of-fit of the network is computed by SEM. SEM is a statistical method to test the hypothesis for the existence of both latent factor-gene and gene-gene interactions. The principal idea of SEM is to minimize the difference between the covariance matrices of the predicted variables and the observed variables. Let Cov(**a, b**) be the covariance matrix of two random vectors **a **and **b**. The LDFM is lag-1 in time, so we consider the joined vector of *y*(*t*)^*T *^and *y*(*t *- 1)^*T*^.

Let **S **denote the sample covariance matrix, which is defined as

$$S=\left[\begin{array}{ll} S_{t,t} & S_{t,t-1}\\ S_{t-1,t} & S_{t-1,t-1} \end{array}\right],$$

where **S**_*t*,*t *_= Cov(**y**(*t*), **y**(*t*)), **S**_*t*-1,*t *_= Cov(**y**(*t - *1), **y**(*t*)), **S**_*t*,*t*-1 _= Cov(**y**(*t*), **y**(*t-*1)), and **S**_*t*-1,*t*-1 _= Cov(**y**(*t *- 1), **y**(*t *- 1)). Let $\hat{y}$(*t*) be the column vector of the predicted expressions for the *n *genes at time *t*. Plugging in $\hat{y}$(*t *- 1) and $\hat{y}$(*t*) for **y**(*t *- 1) and **y**(*t*), respectively into the elements of **S**, we obtain the estimated covariance matrix $\hat{\Sigma }$.

In SSEM, the parameters are estimated by the maximum likelihood method with the fitting function

(4) $$F_{\text{ML}}=\log\left|\hat{\Sigma }\right|-\log\left|S\right|+\text{tr}(S{\hat{\Sigma }}^{-1})-2n,$$

where $\hat{\Sigma }$ denotes the estimated covariance matrix, **S **the sample covariance matrix, |**A**| and tr(**A**) the determinant and the trace of matrix **A**, respectively, and *n *the number of genes. When the sample size (*T*) is small, ridge estimation is applied to avoid the singularity of **S **and $\hat{\Sigma }$. In the application section, small ridge constants are applied such that the condition number of **S **and $\hat{\Sigma }$ are not larger than 10^2^.

Among the six MSC's studied, the *χ*^2 ^statistic is based on the idea of minimizing the discrepancy between the estimated and the sample covariance matrices, and it is defined as (*T *- 1) times the minimized value of *F*_ML _in Equation (4), where *T *is the sample size. When the fitting function is *F*_ML_, the *χ*^2 ^statistic is equivalent to the generalized likelihood ratio [41]. Assuming multivariate normality, the *χ*^2 ^statistic has an asymptotic (large sample) *χ*^2 ^distribution with (p* - *q*) degrees of freedom, where p* = (3*n*^2 ^+ *n*)/2 since only ${\hat{\Sigma }}_{t-1,t}$ and the lower triangle matrix of ${\hat{\Sigma }}_{t,t}$ form equations to estimate parameters. Further, *q *is the number of parameters that equal to *n*^2 ^+ *kn *in the LDFM in (2). The condition *n *> *k *is required to have degrees of freedom p* - *q*> 0. However, this condition is satisfied in general since *k *= [*n/c*], where *c *≥ 3. A large sample size can inflate a small difference between **S **and $\hat{\Sigma }$, and thus can inflate the *χ*^2 ^statistic. Numerous indices were proposed to remedy the bias, among them four have been assessed in our pilot studies, namely, *χ*^2^/*df*, *χ*^2 ^- *df *[42], TLI [43] and CFI [44], where *df *denotes degree of freedom. The former two were found to be more effective than the latter two for network (model) selection.

MSE between the observed and the predicted gene expressions is defined as $\sum_{i=1}^{n}\sum_{t=1}^{T}{\left(y_{i}(t)-{\hat{y}}_{i}(t)\right)}^{2}/nT$, where *T *is the number of time points in the gene expression data. AIC [45] and BIC [46] are two widely used information criteria for model selection, which take model complexity into account. AIC is a measure based on the Kullback-Leibler distance between the fitted and the true model, and AIC = -2log *L*(${\hat{\theta }}_{j}$) + 2*q*_*j*_, where log *L*(${\hat{\theta }}_{j}$) is the log-likelihood with estimates ${\hat{\theta }}_{j}$, and *q*_*j *_is the number of parameters in model *j*. To solve the inconsistency problem of AIC, Schwarz [44] proposed BIC based on maximization of the posterior choice probability. BIC = -2log *L*(${\hat{\theta }}_{j}$) + *q*_*j *_log *T*, where *T *is the number of time points. To reduce the penalty imposed in BIC, Sclove [47] suggested sample-size adjusted BIC (adjBIC) by replacing *T *with T*, where T* = (*T *+ 2)/24.

### Learning networks through iterated SEM

A genetic network inferred from LDFM can be built by latent factor-gene and gene-gene interactions. A correct network is essential for estimation of gene-gene interactions using SEM. However, learning the optimal network from data subject to a goodness-of-fit index is NP-hard. Although global optimization techniques, such as simulated annealing and genetic algorithms, may be applied, the required computation time is not feasible. To make the learning process practical, we propose a stepwise approach. The key idea is to generate a set of candidate networks and retain plausible links by both using SEM and iteratively filtering with a moving window as follows. For any network generated in the iteration, we apply SEM to estimate **x**(*t*), **Λ **and **w**. The significance of each link (*λ*_*ij *_and *W*_*kl*_) is tested by its associated *t*-statistic. Let *t*^*i*^-window (denoted by $\left[t_{l}^{i},t_{u}^{i}\right]$) be a window of some given lower and upper bounds in the *i*th iteration to screen for significance of generated links. A link with a *t*-statistic value greater than $t_{u}^{i}$, within $\left[t_{l}^{i},t_{u}^{i}\right]$ or less than $t_{l}^{i}$ is regarded as a candidate link, a possible link or a futile link (denoted by *c-link*, *p-link *and *f-link*), respectively.

Let *S*_*c*_, *S*_*p *_and *S*_*f *_denote the sets of *c*-links, *p*-links and *f*-links, respectively. Suppose that EFA suggests *k *factors for a given data set. Given fixed *k *- 1, *k *or *k *+ 1 factors, EFA is applied again to learn the associated latent (factor-gene) structures. SSEM begins with the aforementioned latent structure and a fully connected gene structure, namely, each gene is regulated by all genes and *k *- 1, *k *or *k *+ 1 latent factors. To start the stepwise search, SEM is applied to the initial networks to estimate **x**(*t*), **Λ **and **w**. For a given initial network, first let the initial *t*^0^-window be $\left[t_{l}^{0},t_{u}^{0}\right]$. Then, a set of networks Φ^0 ^can be generated as follows. Checking the *t*-statistics of all links against the *t*^0^-window $\left[t_{l}^{0},t_{u}^{0}\right]$, we discard all *f*-links, and retain all *c*-links, while considering all 0–1 combinations of *p*-links. Suppose there are *l p*-links in an initial model, then there are 2^*l *^combinations of each *p*-link being included in a model or not. Models including all *c*-links and each aforementioned combination are considered, and these 2^*l *^models can be viewed as generated by the *t*-window filtering. That is, the *t*-window serves as a filter to eliminate insignificant (*the less-likely-to-exist*) links. Specifically, Φ^0 ^= {*φ*|*φ *∈ *S*_*c *_∪ *L*_*p*_, ∀ *L*_*p *_∈ P(*S*_*p*_)}, where P(*S*_*p*_) = {*L*_*p*_|*L*_*p *_⊆ *S*_*p*_} is the power set of *S*_*p *_and *L*_*p *_is a subset of *S*_*p*_. Furthermore, we apply SEM to each candidate network in Φ^0 ^to obtain the pre-specified goodness-of-fit index. To save computation time and to ensure that the superior networks are kept for the next iteration, only the top *m *networks (denoted by Ω^0^) are retained for the next iteration.

Similar to the initial iteration, for each iteration *i *≥ 1, SSEM generates a set of candidate networks by *t-*window filtering all networks generated by the top *m *networks from iteration(*i*-1), i.e., Ω^*i*-1^, with *k *- 1, *k *or *k *+ 1 factors, respectively, to form Φ^*i*^. So in total, there are *3 m *seed models to generate networks. Among the networks in Φ^*i *^∪ Ω^*i*-1^, only the top *m *networks (Ω^*i*^) are retained by the specified goodness-of-fit index for iteration (*i *+ 1). First, we let the *t*^*i*^-window equal to the *t*^*i*-1^-window +*c*. We use *c *= 0.1, but c can be other small constants. Again, given the *t*^*i*^-window, each link in the *j*th network in Ω^*i*-1 ^can be discarded, retained or considered according to its *t*-statistic value. We denote the collection of these *f*-links, *c*-links, and *p*-links by *S*_*jf*_, *S*_*jc*_, and *S*_*jp*_, respectively. A set of candidate networks is generated by retaining all *c*-links and considering all 0–1 combinations of *p*-links with *k *- 1, *k *or *k *+ 1 factors in the model, and this set is denoted by $\Phi_{j}^{i}=\left\{\varphi |\varphi \in S_{jc}\cup L_{p},\forall L_{p}\in P(S_{jp})\right\}$ = {*φ*|*φ *∈ *S*_*jc *_∪ *L*_*p*_, ∀ *L*_*p *_∈ P(*S*_*jp*_)}. We combine all the generated sets to result in the *i*th set of networks $\Phi^{i}=\underset{\forall j}{\cup }\Phi_{j}^{i}$.

Evaluating the specified goodness-of-fit index for every network in Φ^*i*^, we obtain the top *m *scored networks from Φ^*i *^∪ Ω^*i*-1^, which form Φ^*i*^, to go to iteration (*i *+ 1). The iteration terminates if the specified goodness-of-fit index can not be further improved.

### The proposed SSEM algorithm

#### Initialization

Fit EFA to a given data set to determine the number of factors, say *k*.

• Apply EFA to generate three initial networks by estimating the latent structures with *k *- 1, *k *or *k *+ 1 latent factors, respectively.

• For given k factors, obtain the latent structure by eliminating the links with factor loading less than a constant (the default value used is 0.2).

• Specify an MSC.

#### Stepwise search

• For each initial network:

Step 1. Set iteration *i *= 0, run SEM on the data set. Specify the *t*^0^-window = $\left[t_{l}^{0},t_{u}^{0}\right]$. Generate a set of networks that consist of all *c*-links and one of all the 0–1 combinations of *p*-links. Compute the MSC of all networks and select the top *m *models to form the candidate set Ω^0^.

Step 2. Set *i *= *i *+ 1. Specify the *t*^*i*^-window $[t_{l}^{i},t_{u}^{i}]=[t_{l}^{i-1},t_{u}^{i-1}]+0.1$.

Step 3. Similarly to Step 1, for each network in Ω^*i*-1^, generate a set of networks, and form Φ^*i*^

Step 4. Evaluate the MSC for all networks in Φ^*i*^, and choose the best *m *networks from Φ^*i *^∪ Ω^*i*-1 ^to form the *i*th candidate set Ω^*i*^.

Step 5. If the *i*th top 1 MSC = the (*i*-1)th top 1 MSC, stop ; Otherwise, go to Step 2.

• Select the best *m *networks from the union of all networks generated by different initial guesses.

## Availability and requirements

Project home page is in [48]. SSEM algorithm is written in Visual C++ 6.0, and it calls SAS 8.2 and Mplus 3.0. Program runs under Windows 2000 or higher version operating system. The zipped code of SSEM is attached in Additional file 7. Visual C++, SAS and Mplus are readily available for purchase through Microsoft, SAS and Mplus, respectively.

## Authors' contributions

GS and CC conceived the study, devised the method, and supervised methodology and implementation. CY carried out the method and part of simulation, and wrote an early draft of Shieh et al. in [7]. JH wrote and automated the code. WW participated in implementation. CC and YL wrote part of the paper. GS wrote the paper and coordinated the entire work. All of the authors have read and approved the final manuscript.

## Supplementary Material

Additional file 1 SSEM-TR. Technical Report of SSEM – Shieh et al. (2005). Click here for file

Additional file 2 qRT-PCR. Description of the design of qRT-PCR experiments and how the experiments were conducted to confirm the predicted TC and TD interactions. Click here for file

Additional file 3 Simulation. The description of the 6- and 10-gene networks, and the results of EBVM applied to the two networks. Click here for file

Additional file 4 BayesianNW. The 6-gene network predicted by the three Bayesian network algorithms in Beal et al. (2005), Rangel et al. (2004) and Perrin et al. (2003). Click here for file

Additional file 5 Multi-AR(1). The result of fitting multivariate AR(1) straightforwardly to the real data for the 6-gene network. Click here for file

Additional file 6 SSL-TCNW. TC networks of SSL gene pairs. Description of how TC and TD interactions of SGS1 and RAD27 SSL gene pairs were predicted by SSEM. Click here for file

Additional file 7 SSEM-algorithm. The zipped file consists of the standalone executable (.exe) file of SSEM. Click here for file
