# Supplementary material for: Inferring transcriptional compensation interactions in yeast via stepwise structure equation modeling
Source: BMC Bioinformatics. 2008 Mar 3;9:134. doi: 10.1186/1471-2105-9-134 (PMC2323972; doi:10.1186/1471-2105-9-134)
Supplement: Additional file 1 — SSEM-TR. Technical Report of SSEM – Shieh et al. (2005). [file 1471-2105-9-134-S1.pdf]

Systems biology

A Stepwise Structural Equation Modeling Algorithm to Reconstruct Genetic Networks

Grace S. Shieh[1,*,†], Chung-Ming Chen[2,*], Ching-Yun Yu[1], Juiling Huang[1] and Woei-Fuh Wang[2]

[1] Institute of Statistical Science, Academia Sinica, Taipei, Taiwan

[2] Institute of Biomedical Engineering, National Taiwan University, Taipei, Taiwan

ABSTRACT

Motivation: A stepwise structural equation modeling algorithm (SSEM) has been developed to infer genetic networks from time course microarray data. Besides observed variables, SSEM also incorporates hidden variables in order to depict regulations from proteins and other molecules that are not measured by microarrays. SSEM can learn the structure of a genetic network from microarray data. We have simulated data from a 6-gene and a 10-gene network, under median to high noise levels, to determine with which criterion SSEM works best out of the six goodness-of-fit indices studied. Next, we have applied SSEM to real microarray data in yeast (with no replicates) to infer transcriptional compensation interactions among six genes.

Results: SSEM with BIC results in the highest true positive rates, the largest percentage of correctly predicted links from the total number of existing links, and the highest true negative (non-existing links) rates. We have applied SSEM with BIC to reconstruct a 6-gene network in yeast, and compared the results with those obtained using three Bayesian network algorithms from Beal *et al*. (2005), Rangel *et al*. (2004) and Perrin *et al*. (2003). The modified true positive rates of SSEM and the former two algorithms are about 56%, 13% and 44%, respectively; while LDS of Perrin *et al*. (2003) can not confirm the existence of any links.

Contact: gshieh@stat.sinica.edu.tw

Availability: Supplementary data is available at http://www.stat.siniac.edu.tw/~gshieh/ssem.htm.

1 INTRODUCTION

Gaining an understanding of genetic interactions to unravel the mechanisms of various biological processes in living cells has been a longtime endeavor. Due to the fragmentary knowledge of the genomes and the limited capability of experimental tools, for decades, biologists were limited to delving into a small number of genes at a time using costly and time-consuming procedures. This situation has changed greatly with the emergence of modern biotechnologies. High-throughput DNA sequencing technologies have led to the genomes of several species being completely sequenced, which paves the way for further studies on all information encoded in the genetic blueprint. In addition, the advanced microarray technologies offer the power to observe the expression levels of tens of thousands of genes in an organism simultaneously. Microarray data not only reveal which genes are involved in a biological process, which might have required a few years to identify using conventional laboratory approaches, but also provide the opportunity to infer the genetic regulations among a group of genes.

With the abundant information produced by microarray technology, various approaches have been proposed to infer genetic regulatory networks. Most of them may be classified into three classes, namely, discrete variable models, continuous variable models and graph models. The discrete variable models discretize gene expressions into a few states. The dynamics of gene expressions may be perceived as transitions of finite states. Typical discrete variables models proposed are Boolean networks (Liang *et al.*, 1998; Akutsu *et al.*, 1999; Akutsu *et al.*, 2003), probabilistic Boolean networks (Shmulevich, Dougherty, Kim *et al.*, 2002; Shmulevich, Dougherty, and Zhang, 2002), reversible-jump Markov-Chain Monte Carlo (MCMC) predictor design (Zhou *et al.*, 2003) and discrete Bayesian networks, namely, Bayesian networks with discrete variables (Friedman *et al.*, 2000; Hartemink *et al.*, 2002). Both Boolean networks and probabilistic Boolean networks simplify the gene expressions into two states, i.e., on or off, and characterize genetic regulations by logic rules. The logic function at each node of a Boolean network is fixed, while that of a probabilistic Boolean network changes with time and is determined probabilistically. The reversible-jump MCMC predictor design (Zhou *et al.*, 2003) further extends the binary nature of the probabilistic Boolean network to a multiple-state model with non-Boolean predictor function. While the first three classes of networks model genetic regulation using rules, discrete Bayesian networks characterize gene-gene regulations using a joint distribution. The optimal discrete Bayesian network is determined by a Bayesian scoring metric. The discrete variable models have advantages in being robust to noises and requiring a small amount of data. However, these advantages are attained at the cost of inducing potential errors due to

[*] These authors contributed equally to this work.

[†] To whom correspondence should be addressed.

discretizing intrinsically continuous gene expressions into a number of states.

In general, continuous variable models characterize the expression of a gene or its change by a linear or non-linear continuous function of the expression of other genes. The gene-gene regulations are frequently modeled by a first-order or a second-order differential (or difference) equation. Continuous variable models fall into two major classes. One is continuous Bayesian networks (Perrin *et al.*, 2003; Tamada *et al.*, 2003; Beal *et al.*, 2005; Zou and Conzen, 2005), i.e., Bayesian networks with continuous variables, and the other is deterministic differential systems (Ando *et al.*, 2002; Kikuchi *et al.*, 2003; Thomas *et al.*, 2004; Guthke *et al.*, 2005; Kimura *et al.*, 2005). Continuous Bayesian networks assume a causal relation with the regulations, which follow conditional probability distributions. The entire system of causal relations among genes is modeled as a joint distribution of all genes in the network. Continuous Bayesian networks are appealing for deciphering genetic regulation because of their solid basis in statistics, which allows them to handle the stochastic aspects of noisy microarray data and incomplete data. However, continuous Bayesian networks suffer from two drawbacks. First, the parameters involved require much more data than other approaches. Second, their performance may be seriously compromised if the assumed joint distribution is far from the underlying distribution.

Unlike continuous Bayesian networks, which assume a causal relation between two genes, deterministic differential systems, in general, postulate that the gene-gene relation is governed by a deterministic first- or second-order differential (or difference) equation with or without noise, such as an S-system (Kikuchi *et al.*, 2003; Kimura *et al.*, 2005). In comparison with continuous Bayesian networks, deterministic differential systems generally require less data. In theory, when using continuous data, deterministic differential systems characterize genetic interactions more realistically than Boolean networks and graph models. Nevertheless, the data produced by most microarray experiments are often still insufficient to compute the unknowns in a deterministic differential system. Furthermore, owing to the deterministic nature, the resulting networks are not robust to noise of gene expressions generated by the stochastic processes in a living organism.

Graph models (Wagner, 2001; de la Fuente *et al.*, 2004; Kyoda *et al.*, 2004; Schäfer and Strimmer, 2005) depict gene-gene regulations through directed graphs or digraphs instead of characterizing the regulations quantitatively. They offer the simplest representation of a genetic regulation among all three classes of network models. Some graph models simply reveal structural information, others annotate the directions and signs of the regulations among genes. Because of their simplicity, graph models usually require much less data than models in the other two categories. But they are inherently static and may not capture the dynamics of genetic regulations and the simultaneous regulation of a given gene by multiple genes.

Although each class of models has been shown to be informative for understanding various levels of gene regulations, most of the models proposed have the overfitting problem with the exception of some Bayesian networks. The overfitting problem arises from the fact that microarrays measure the mRNA expressions only, while genetic interactions may be regulated by enzymes or proteins, for instance transcriptional factors, which are not measured by microarrays. Furthermore, most genetic networks reconstructed in previous studies considered only a subset of the whole genome. Consequently, those genes that wre left out may be regarded as latent factors regulating the genes of interest. Thus ignoring latent factors in the models may cause bias on inferring the genetic regulations. Although a Bayesian network can incorporate latent factors (Perrin *et al.*, 2003; Beal *et al.*, 2005), the amount of data required may prevent it from being used in practice.

To account for the latent factors effect using a reasonable amount of microarray data, a stepwise structural equation modeling algorithm (SSEM) is proposed. SSEM is based on structural equation modeling (SEM) (Kaplan, 2000) which unifies factor analysis and path analysis. Assuming linear relations among the observed and latent variables, the basic idea of SEM is to minimize the discrepancy between the fitted covariance matrix and the sample covariance matrix. Although path analysis was introduced in the early 1900s (Wright, 1918), SEM has not received attention tantamount to its versatility. Thus far, inferring genetic interactions using SEM can only be found in Xie and Bentler (2003). They showed that the latent factors can be identified and their relations may be estimated reasonably by SEM. However, it was not demonstrated how to capture the causal relations among genes, neither the dynamics of regulations among genes. Note that without identifying the latent factors reasonably, the causal relations among genes can not be estimated correctly, and vice versa; SSEM attempts to estimate latent factors involved and causal regulations among genes simultaneously. The basic idea is to learn genetic regulations by both exploratory factor analysis (EFA) and SEM with various model selection criteria in a stepwise fashion. The performance of SSEM is evaluated using data from two simulated genetic networks. Following a gene's loss, its compensatory gene's expression increases, this phenomenon is called *transcriptional compensation* (Wong and Roth, 2005). While the existence of genetic compensation is well received, the mechanism is still unknown. We apply SSEM to the yeast microarray data in Spellman *et al.* (1998) to infer transcriptional compensation interactions of six genes, among which *SGS1* is synthetic sick or lethal to the other five (Tong *et al.*, 2001; Tong *et al.*, 2004).

2 SYSTEMS AND METHODS

The linear dynamic factor model

We assume that time course microarray data follow a linear dynamic factor model (LDFM), which includes both factor-gene and gene-gene regulation in the model. Let $y_i(t)$ denote the expression of gene i at time t for $1 \le i \le n$, where n is the number of genes in the network. Specifically, LDFM assumes that $y_i(t)$ is regulated by a linear combination of latent factors at time t and observed variables (genes) at time $(t-1)$, and the regulation is invariant across time as follows:

$$\mathbf{y}(t) = \mathbf{\Lambda x}(t) + \mathbf{Wy}(t-1) + \boldsymbol{\varepsilon} \qquad (1)$$

where $\mathbf{y}(t)$ is the $(n \times 1)$ vector of the expression levels of the n genes at time *t*, $\mathbf{x}(t)$ is the $(k \times 1)$ vector of the latent factors at time *t*, $\mathbf{\Lambda}$ is the $(n \times k)$ latent interaction matrix, in which λ_{ij} denotes the influence of latent factor *j* on the expression of gene *i* at the same time, $\mathbf{W}$ is the gene-gene interaction matrix, in which ω_{ij} denotes the influence of the expression of gene *j* at time $(t-1)$ on that of gene *i* at time *t*, and $\boldsymbol{\varepsilon}$ is the $(n \times 1)$ output noise vector that assumes $N(0,Q)$, where Q is the covariance matrix. The LDFM is illustrated in Figure 1.

Figure 1 ABOUT HERE

The major difference between LDFM and the state space model (SSM), a variation of the continuous Bayesian networks in Perrin *et al.* (2003) and Beal *et al*. (2005), is that the former does not model interactions among latent factors across time, while the latter does. Thus LDFM requires less data, and is feasible when limited replicates of microarray experiment are available.

An SSEM algorithm is proposed to learn the parameters $\mathbf{x}(t)$, $\mathbf{\Lambda}$ and $\mathbf{W}$ in LDFM. The main idea is to learn the regulation network iteratively. In each iteration, for every network generated, we estimate the parameters by SEM and evaluate the goodness-of-fit. The top few networks in each iteration are retained for the next iteration, until the optimal networks, in terms of any goodness-of-fit index (GFI), emerge.

In Part 2, networks composed of any given initial latent structure and a fully connected gene-gene interaction structure are considered. SEM is applied to estimate the parameters $\mathbf{x}(t)$, $\mathbf{\Lambda}$ and $\mathbf{W}$ of any network considered, and a specified model selection criterion evaluates the goodness-of-fit of the network. In Part 3, plausible networks are generated by systematically and iteratively eliminating insignificant links based on the associated *t*-statistics resulting from SEM. These three parts are described in details in the learning networks section.

Learning the initial latent structures

Incorporating a correct latent structure is crucial for reconstructing genetic networks. One way to learn the latent factors is to try different numbers of latent factors and choose the optimal number based on a cost function as in Beal *et al*. (2005). While this promises a comprehensive search for the latent factors involved, it is inefficient since most trials are redundant. Moreover, it provides no clues for finding the latent structures. Alternatively, EFA is employed in Part 1 of SSEM to learn potential latent structures to start the iterative process.

EFA is a common practice to ascertain the latent factors that influence the observed variables. Fundamentally, factor analysis assumes that there are some latent factors, fewer in number than genes, that are responsible for the co-variation among the observed gene expressions. EFA may be expressed as

$$\mathbf{y}(t) = \tilde{\mathbf{\Lambda}}\tilde{\mathbf{x}}(t) + \tilde{\mathbf{u}}, \qquad (2)$$

where $\tilde{\mathbf{x}}(t)$, $\tilde{\mathbf{\Lambda}}$ and $\tilde{\mathbf{u}}(t)$ are all estimated without taking gene-gene interaction into account. Specifically, $\tilde{\mathbf{x}}(t)$ is $(m \times 1)$ the common factors at time *t*, $\tilde{\mathbf{\Lambda}}$ is the $n \times m$ latent interaction matrix, in which λ_{ij} denotes the influence of latent factor *j* on the expression of gene *i* at time *t* estimated without explicitly taking account of gene-gene interaction, and $\tilde{\mathbf{u}}(t)$ is $(m \times 1)$ the unique factors at time *t* that can not be explained by the common factors $\tilde{\mathbf{x}}(t)$. Comparing Equations (1) and (2), the latent structure embedded in $\tilde{\mathbf{\Lambda}}\tilde{\mathbf{x}}(t)$ would deviate from the true one except when the factor $\tilde{\mathbf{u}}(t)$ accounts for the effect of gene-gene interaction, that is, equal to $Wy(t-1)$. This shows that fitting the gene expressions to a structural equation model with the latent factors estimated solely by EFA (Xie and Bentler, 2003) may not result in correct latent structure. Therefore, supposing that *k* latent factors are suggested by EFA, we consider $k-1$, *k* or $k+1$ three possible numbers of latent factors, along with the associated latent structure in Part 1 of SSEM. The common factors are extracted by a principal component analysis with *promax* oblique rotation.

Determining the number of common factors that best explain the observed variables is one of the practical issues in exploratory factor analysis. Various guidelines have been proposed, for instance, eigenvalue ≥ 1 (Guttman, 1954) and the scree test (Cattell, 1966). Different guidelines may lead to different choices. Based on the "weaker lower bound" suggested by Guttman (1954), SSEM seeks through $k-1$, *k*, and $k+1$ common factors and the associated latent structures, where *k* is the number of common factors with eigenvalues ≥ 1 resulting from EFA. Then, for each given number of common factors, the latent structure is obtained by eliminating the links with factor loading less than 0.4.

Network (model) selection criterion

In the iterations of SSEM, the parameters $\mathbf{x}(t)$, $\mathbf{\Lambda}$ and $\mathbf{W}$ of a given network are estimated, and the goodness-of-fit of the network is assessed by SEM. SEM is a statistical method to test the hypothesis for the existence of both latent factor-gene and gene-gene interactions. The principal idea of estimating parameters in SEM is to minimize the difference between the covariances of the predicted variables and the observed variables. Let $Cov(\mathbf{a},\mathbf{b})$ be the covariance matrix of two random vectors **a** and **b**. The LDFM is lag-1 in time, so we consider the joined vector of $y(t)^T$ and $y(t-1)^T$. Let $\mathbf{S}$ denote the sample covariance matrix, which is defined as

$$\mathbf{S} = \begin{bmatrix} \mathbf{S}_{t,t} & \mathbf{S}_{t,t-1} \\ \mathbf{S}_{t-1,t} & \mathbf{S}_{t-1,t-1} \end{bmatrix},$$

where $\mathbf{S}_{t,t} = \text{Cov}(\mathbf{y}(t),\mathbf{y}(t))$, $\mathbf{S}_{t-1,t} = \text{Cov}(\mathbf{y}(t-1),\mathbf{y}(t))$, $\mathbf{S}_{t,t-1} = \text{Cov}(\mathbf{y}(t),\mathbf{y}(t-1))$, and $\mathbf{S}_{t-1,t-1} = \text{Cov}(\mathbf{y}(t-1),\mathbf{y}(t-1))$. Let $\hat{\mathbf{y}}(t)$ be the column vector of the predicted expressions for the n genes at time *t*. Similarly, let $\hat{\mathbf{\Sigma}}$ denote the estimated covariance matrix defined as

$$\hat{\mathbf{\Sigma}} = \begin{bmatrix} \hat{\mathbf{\Sigma}}_{t,t} & \hat{\mathbf{\Sigma}}_{t,t-1} \\ \hat{\mathbf{\Sigma}}_{t-1,t} & \hat{\mathbf{\Sigma}}_{t-1,t-1} \end{bmatrix},$$

where $\hat{\mathbf{\Sigma}}_{t,t} = \text{Cov}(\hat{\mathbf{y}}(t),\hat{\mathbf{y}}(t))$, $\hat{\mathbf{\Sigma}}_{t-1,t} = \text{Cov}(\hat{\mathbf{y}}(t-1),\hat{\mathbf{y}}(t))$,

$\hat{\boldsymbol{\Sigma}}_{t,t-1} = \mathrm{Cov}(\hat{\mathbf{y}}(t), \hat{\mathbf{y}}(t-1))$, and $\hat{\boldsymbol{\Sigma}}_{t-1,t-1} = \mathrm{Cov}(\hat{\mathbf{y}}(t-1), \hat{\mathbf{y}}(t-1))$.

In SSEM, the parameters are estimated by the maximum likelihood method with the fitting function,

$$F_{\mathrm{ML}} = \log\left|\hat{\boldsymbol{\Sigma}}\right| - \log\left|\mathbf{S}\right| + \mathrm{tr}(\mathbf{S}\hat{\boldsymbol{\Sigma}}^{-1}) - p, \qquad (3)$$

where $\hat{\boldsymbol{\Sigma}}$ denotes the estimated covariance matrix, $\mathbf{S}$ the sample covariance matrix , $|\mathbf{A}|$ and $\mathrm{tr}(\mathbf{A})$ the determinant and the trace of matrix $\mathbf{A}$, respectively, and p the number of genes.

Various indices have been proposed to assess the goodness-of-fit of a structural equation model. While different goodness-of-fit indices stress on different model properties, the best index for genetic network reconstruction remains unknown. Rather than resort to a specific goodness-of-fit index, we have carried out a large-scale simulation to evaluate eight indices used in commercial SEM softwares, such as SAS 8.02 (SAS Institute, 1988) and Mplus version 3 (Muthén & Muthén, 2004). Based on our pilot studies which examined eight indices, the performances of the top six are reported here: two modified χ^2 statistics, namely, χ^2/df and $\chi^2 - df$ (Jöreskog, 1969), where df denotes degree of freedom; mean square error (MSE) between the observed and the predicted gene expressions; Akaike information criterion (AIC) (Akaike, 1973); Bayesian information criterion (BIC) (Schwarz, 1978), and sample-size adjusted BIC (adjBIC) (Sclove, 1987).

Based on the idea of minimizing the discrepancy between the estimated and the sample covariance matrices, the T statistic has been widely used as a goodness-of-fit index. The T statistic is defined as $(N-1)$ times the minimized value of F_{ML} in Equation (3), where N is the sample size. When the fitting function is F_{ML}, the T statistic is equivalent to the generalized likelihood ratio (Kline, 1998). Assuming multivariate normality, the T statistic has an asymptotic (large sample) χ^2 distribution with $(p^* - q)$ degrees of freedom, where $p^* = p(p+1)/2$ and q is the number of parameters. A large sample size can inflate a small difference between $\mathbf{S}$ and $\hat{\boldsymbol{\Sigma}}$, and thus can inflate the T statistic. Numerous fit indices were proposed to remedy the bias, among them four have been assessed in our pilot studies, namely, χ^2/df , $\chi^2 - df$ (Jöreskog, 1969), TLI (Tucker and Lewis, 1973) and CFI (Bentler, 1990). The former two were more effective than the latter two for network (model) selection via SEM in our pilot studies.

MSE is defined as

$$\sum_{i=1}^{n}\sum_{t=1}^{T}(y_i(t) - \hat{y}_i(t))^2 \big/ nT,$$

where T is the number of time points in the gene expression data. AIC and BIC are two widely used information criteria for model selection, which take model complexity into account. AIC is a measure based on the Kullback-Leibler distance between the fitted and the true model, and it has the form

$$\mathrm{AIC} = -2\log L(\hat{\boldsymbol{\theta}}_j) + 2q_j,$$

where $\log L(\hat{\boldsymbol{\theta}}_j)$ is the log-likelihood with estimates $\hat{\boldsymbol{\theta}}_j$, and q_j is the number of parameters in model j. The first term of AIC measures model fit, whereas the second penalizes on model complexity (the addition of parameters). To solve the inconsistency problem of AIC, Schwarz (1978) proposed Bayesian information criterion (BIC) based on maximization of the posterior choice probability. BIC assumes the form

$$\mathrm{BIC} = -2\log L(\hat{\boldsymbol{\theta}}_j) + 2q_j \log N,$$

where $N = nT$. To reduce the penalty imposed in BIC, Sclove (1987) suggested a sample size adjustment by replacing N with N , where $N^* = (N+2)/24$.

Learning networks through iterated SEM

A genetic network inferred from LDFM can be built by latent factor-gene and gene-gene interactions. A correct network is essential for estimation of gene-gene interactions using SEM. However, learning the optimal network from data subject to a goodness-of-fit index is an NP-hard problem. Although global optimization techniques, such as simulated annealing and genetic algorithm, may be applied, the required computation time is not desirable. To make the learning process practical, we propose a stepwise approach. The key idea is to generate a set of candidate networks and retain plausible links by both using SEM and iteratively filtering with a moving window as follows. For any network generated in the iteration, we apply SEM to estimate $\mathbf{x}(t)$, $\boldsymbol{\Lambda}$ and $\mathbf{W}$. The significance of each link (λ_{ij} and ω_{kl}) is tested by its associated t-statistic. Let t^i-window (denoted by $[t_l^i, t_u^i]$) be a window of some given lower and upper bounds in the ith iteration to screen for significance of generated links. A link with a t-statistic value greater than t_u^i , within $[t_l^i, t_u^i]$ or less than t_l^i is regarded as a candidate link, a possible link or a futile link (denoted by *c-link*, *p-link* and *f-link*), respectively. Furthermore, let S_c , S_p and S_f denote the sets of c-links, p-links and f-links, respectively.

Suppose that EFA suggests k factors for a given data set. Given fixed $k-1$, k or $k+1$ factors, EFA is applied again to learn the associated latent (factor-gene) structures. SSEM begins with the aforementioned latent structure and a fully connected gene structure, namely, each gene is regulated by all genes and $k-1$, k or $k+1$ latent factors. To start the stepwise search, SEM is applied to the initial networks to estimate $\mathbf{x}(t)$, $\boldsymbol{\Lambda}$, and $\mathbf{W}$. For a given initial network, first let the initial t^0-window be $[t_l^0, t_u^0]$. Then, a set of networks Φ^0 can be generated as follows. Checking the t-statistics of all links against the t^0-window $[t_l^0, t_u^0]$, we discard all f-links, and retain all c-links, while considering all 0-1 combinations of p-links. Suppose there are l p-links in an initial model, then there are 2^l combinations of each p-link being included in a model or not. Models including all c-links and each aforementioned combination are considered, and these 2^l models can be viewed as generated by the t-window filtering. That is, the t-window serves as a filter to eliminate insignificant (*the less-likely-to-exist*) links. Specifically, $\Phi^0 = \left\{\phi \mid \phi \in S_c \cup L_p, \forall L_p \in \mathrm{P}(S_p)\right\}$, where $\mathrm{P}(S_p) = \left\{L_p \mid L_p \subseteq S_p\right\}$ is the power set of S_p and L_p is a subset

of S_p. Furthermore, we apply SEM to each candidate network in Φ^0 to obtain the pre-specified goodness-of-fit index. To save computation time and to ensure that the superior networks are kept for the next iteration, only the top *m* networks (denoted by Ω^0) are retained for the next iteration.

Similar to the initial iteration, for each iteration $i \geq 1$, SSEM generates a set of candidate networks by *t*-window filtering all networks generated by the top *m* networks from iteration (*i*-1), i.e., Ω^{i-1}, with $k-1$, k or $k+1$ factors, respectively, to form Φ^i. So in total, there are *3 m* seed models to generate networks. Among the networks in $\Phi^i \cup \Omega^{i-1}$, only the top *m* networks (Ω^i) are retained by the specified goodness-of-fit index for iteration $(i+1)$. First, we let the t^i-window equal to the t^{i-1}-window $+c$. We use $c = 0.1$, but c can be other small constants. Again, given the t^i-window, each link in the *j*th network in Ω^{i-1} can be discarded, retained or considered according to its *t*-statistic value. We denote the collection of these *f*-links, c-links, and p-links by S_{jf}, S_{jc}, and S_{jp}, respectively. A set of candidate networks is generated by retaining all c-links and considering all 0-1 combinations of *p*-links with $k-1$, k or $k+1$ factor in the model, and this set is denoted by $\Phi_j^i = \{\phi \mid \phi \in S_{jc} \cup L_p, \forall L_p \in \mathrm{P}(S_{jp})\}$. We union all generated sets to result in the *i*th set of networks $\Phi^i = \bigcup_{\forall j} \Phi_j^i$. Evaluating the specified goodness-of-fit index for every network in Φ^i, we obtain the top *m* scored networks from $\Phi^i \cup \Omega^{i-1}$, which form Ω^i, to pass to iteration (*i*+1). The iteration terminates if the specified goodness-of-fit index can not be further improved.

The proposed SSEM algorithm

Initialization

Fit EFA to a given data set to determine the number of factors, say *k*.

- Apply EFA to generate three initial networks by estimating the latent structures with $k-1$, k or $k+1$ latent factors, respectively.
- Specify a GFI.

Stepwise Search

- For each initial network:
  - Step 1. Set iteration *i* = 0, run SEM on the data set. Specify the t^0-window = $[t_l^0, t_u^0]$. Generate a set of networks that consist of all *c*-links and one of all the 0-1 combinations of *p*-links. Compute the GFI of all networks and select the top *m* models to form the candidate set Ω^0.
  - Step 2. Set $i = i+1$. Specify the t^i-window $[t_l^i, t_u^i] = [t_l^{i-1}, t_u^{i-1}] + 0.1$.
  - Step 3. Similarly to Step 1, for each network in Ω^{i-1}, generate a set of networks, and form $\Phi^i = \bigcup_{\forall j} \Phi_j^i$.
  - Step 4. Evaluate the GFI for all networks in Φ^i, and choose the best m networks from $\Phi^i \cup \Omega^{i-1}$ to form the *i*th candidate set Ω^i.
  - Step 5. If the *i*th top 1 GFI = the (*i*-1)th top 1 GFI, stop ;
    Otherwise, go to Step 2.
- Select the best *m* networks from the union of all networks generated by different initial guesses.

3 IMPLEMENTATION

In this section, we first use simulated gene expression data to evaluate the performance of SSEM with various goodness-of-fit indices. Time course data from both a 6-gene and a 10-gene network are simulated. Next, SSEM is applied to a set of real time course microarray data to reconstruct a 6-gene network. Since the network topology, latent factors ($\mathbf{x}(t)$), gene-gene regulations ($\mathbf{W}$), and latent factor-gene regulations ($\mathbf{\Lambda}$) are well defined for the simulated data, exact quantitative performance can be accessed. The goal is to determine with which index among χ^2/df, $\chi^2 - df$, MSE, AIC, BIC, and adjBIC, SSEM performs best under various numbers of genes, sample sizes and noise levels. On the other hand, real microarray data contain true genetic interactions that may not be revealed in simulated networks. To see how SSEM performs relative to Bayesian networks, we apply SSEM and three Bayesian network algorithms from Beal *et al*. (2005), Rangel *et al.* (2004) and Perrin *et al*. (2003) to reconstruct a network that's regulations were confirmed by biological experiments.

Simulated time course data

Two genetic regulatory networks are simulated; a 6-gene network and a 10-gene network, and both have two latent factors. Experiences from the social sciences indicate that SEM works well provided that the ratio of observed variables to latent factors is at least 3, and our pilot studies also confirmed this. When the ratio of observed variables to latent factors is 5:2, the convergent solutions of the MLEs was only 58% for *T* = 50 and 81% for *T* = 100; but when the ratio is 6:2, the convergent solutions increased to 73% and 93% for *T* = 50 and 100, respectively. We adopted this 3 or higher ratios in the simulation studies. The linear dynamic factor model for the 6-gene network is defined as:

$$\begin{aligned}
y_1(t) &= 0.5x_1(t) + 0.5y_1(t-1) + 0.6y_2(t-1) + \varepsilon_1(t) \\
y_2(t) &= 0.7x_1(t) + 0.5y_2(t-1) + 0.4y_3(t-1) + \varepsilon_2(t) \\
y_3(t) &= 0.7x_1(t) + 0.5y_3(t-1) + 0.4y_4(t-1) + 0.5y_5(t-1) + \varepsilon_3(t) \\
y_4(t) &= 0.6x_2(t) + 0.6y_5(t-1) + \varepsilon_4(t) \\
y_5(t) &= 0.7x_2(t) + 0.5y_5(t-1) + \varepsilon_5(t) \\
y_6(t) &= 0.5x_2(t) + 0.5y_4(t-1) + 0.4y_6(t-1) + \varepsilon_6(t)
\end{aligned}, \tag{4}$$

where $x_1(t) \sim N(0,0.1)$, $x_2(t) \sim N(0,0.1)$, $y_i(0) \sim U(0,1)$, and $\varepsilon_i(t) \sim N(0,\sigma_i^2)$, $i = 1,\ldots,6$. Note that σ_i^2 is determined by the variance of $y_i(t)$ and a pre-specified noise level. The noise level is quantified by a contrast-to-noise ratio (CNR), defined as the ratio

of the signal standard deviation to the noise standard deviation. *CNR* = 1.3 or 2.0 correspond to high or median noise levels, respectively. Similarly, the linear dynamic factor model for the 10-gene network is defined as follows.

$$\begin{aligned}
y_1(t) &= 0.7x_1(t) + 0.5y_1(t-1) + 0.45y_2(t-1) + \varepsilon_1(t) \\
y_2(t) &= 0.75x_1(t) + 0.5y_2(t-1) + 0.4y_3(t-1) + \varepsilon_2(t) \\
y_3(t) &= 0.7x_1(t) + 0.45y_3(t-1) + 0.5y_4(t-1) + \varepsilon_3(t) \\
y_4(t) &= 0.7x_1(t) + 0.45y_4(t-1) + 0.5y_5(t-1) + \varepsilon_4(t) \\
y_5(t) &= 0.85x_1(t) + 0.75y_5(t-1) + \varepsilon_5(t) \\
y_6(t) &= 0.7x_2(t) + 0.5y_6(t-1) + 0.45y_7(t-1) + \varepsilon_6(t) \\
y_7(t) &= 0.75x_2(t) + 0.5y_7(t-1) + 0.4y_8(t-1) + \varepsilon_7(t) \\
y_8(t) &= 0.7x_2(t) + 0.45y_8(t-1) + 0.5y_9(t-1) + \varepsilon_8(t) \\
y_9(t) &= 0.7x_2(t) + 0.45y_9(t-1) + 0.5y_{10}(t-1) + \varepsilon_9(t) \\
y_{10}(t) &= 0.85x_2(t) + 0.75y_{10}(t-1) + \varepsilon_{10}(t),
\end{aligned} \tag{5}$$

where $x_1(t) \sim N(0,0.1)$, $x_2(t) \sim N(0,0.1)$, $y_i(0) \sim U(0,1)$, and $\varepsilon_i(t) \sim N(0,\sigma_i^2)$, $i = 1,\ldots,10$. σ_i^2 is set by the variance of $y_i(t)$, and *CNR* = 1.3 or 2.0. As an example, Figure 2 illustrates the dynamics of the 6-gene network at time *t*, that is, the gene expressions at time *t* are regulated by the latent factors at time *t* and the gene expressions at time $t-1$. Note that for each *i*, figures of the first 100 time points and the last 100 time points of $y_i(t)$ with *T* = 1000 show sinusoid patterns, and can be obtained from the authors upon request.

Figure 2 ABOUT HERE

Note that the networks in (4) and (5) are sparse which roughly follow the sparse property of *cis*-regulatory networks stated in van Someran *et al.* (2002). For each network in (4) and (5), time course data were simulated under four different conditions; sample sizes (*T = 50* or *100*) and noise levels (high or median), where *T* is the number of time points. For the 6-gene network and a given set of (*CNR*, *T*), 100 experiments were carried out, and each simulation took about six days.

Tables 1 and 2 summarize the performance of SSEM with the six goodness-of-fit indices when (*CNR*, *T*) = (2, 100) and (2, 50), respectively. The averages of the true positive rate (TPR), true negative rate (TNR), and false positive rate (FPR) of all top 1 (top 5) networks, in terms of the goodness-of-fit index value, in 100 experiments are reported. TPR (also known as sensitivity) is the percentage of correctly predicted links from the total existing links (positives) in the simulated network. Likewise, TNR (specificity) is the percentage of correctly predicted non-existing links (negatives) out of the total non-existing links in the simulated network.

Taking 5% random error into account, SSEM with BIC clearly performs better than SSEM with the other five goodness-of-fit indices when (*CNR*, *T*) = (2, 100) and (2, 50). The TPRs and TNRs of SSEM with BIC are much higher than those of SSEM with adjBIC, MSE, $\chi^2 - df$ or χ^2 / df. TPRs and TNRs of SSEM with BIC range from 95% to 97% when (*CNR*, *T*) = (2, 100), and range from 84% to 88% when (*CNR*, *T*) = (2, 50). Both SSEM with $\chi^2 - df$ and with χ^2 / df tend to have high sensitivities (TPRs) but low specificities (TNRs) when (*CNR*, *T*) = (2, 100) and (2, 50). Similar observation may be made for SSEM with adjBIC when (*CNR*, *T*) = (2, 50). SSEM with MSE has the worst performance since its TPRs and TNRs ranging from 69% to 77% when (*CNR*, *T*) = (2, 50) though it performs similarly to SSEM with χ^2 / df when (*CNR*, *T*) = (2, 100). Note that the TNR of `Top 1 model' resulted from SSEM with MSE is significantly lower than that of `Top 5 models' for data generated from the 6-gene network with *CNR* = 2. This shows that assigning no penalty on model complexity of MSE causes an over-fitting problem, namely, a network that has more links tends to have a better MSE score but it may not be closer to the true network.

Table 1 ABOUT HERE

Table 2 ABOUT HERE

Though their performances are close and they clearly outperform the other four, for all four cases, SSEM with BIC results in higher TNRs than SSEM with AIC, taking 5% random error into account. Due to the larger penalty on adding a link, BIC is more stringent than AIC in including a link in the network because BIC has significantly higher TNRs (namely lower FPRs). We further compare SSEM with BIC and SSEM with AIC under (*CNR*, *T*) = (1.3, 100) and (1.3, 50), respectively; the results are in Table 3. When CNR decreases from 2 to 1.3 (data are noiser), TPRs and TNRs of both SSEMs deteriorate about 10% for both sample sizes. However, BIC remains superior to AIC. Furthermore, even with a high noise level (*CNR* = 1.3), SSEM with BIC still results in TPRs and TNRs about 85% and 89%, respectively for *T* = 100, and about 73% and 82%, respectively for *T* = 50.

Table 3 ABOUT HERE

The simulation studies on the 6-gene network show that SSEM with BIC performs relatively well to very well under the four cases. To see how network complexity influences SSEM, we ran SSEM with BIC on data simulated from the 10-gene network in (5), with 50 experiments under the four cases of (*CNR*, *T*). The simulation results are summarized in Table 4. In general, SSEM with BIC performances on the 10-gene network are not significantly different from those on the 6-gene network for all cases. For the 10-gene network, TPRs (TNRs) of the Top 1 model resulted from SSEM with BIC are about 98%, 86%, 82%, and 73% (97%, 90%, 88% and 87%), for (CNR, *T*) = (2, 100), (2, 50), (1.3, 100), and (1.3, 50), respectively.

Table 4 ABOUT HERE

Real time course microarray data

Following a gene's loss, its compensatory gene's expression increases, this phenomenon is called *transcriptional compensation.* Typical indicators of a compensatory relationship are redundant genes (paralogs), redundant pathways, and synthetic sick or lethal (SSL) interactions (Wong and Roth, 2005). Since the mechanism of transcriptional compensation is largely unknown, it is of interest to our collaborators in biochemistry whether gene network reconstruction algorithms can help infer such genetic interactions. In this section, SSEM and three Bayesian network algorithms are applied to real microarray data to infer the genetic network in Figure 3, in which the existing links were confirmed by RT-PCR experiments.

Figure 3 ABOUT HERE

The *SGS1* gene is SSL with each one of the rest five genes (Tong *et al*., 2001; Tong *et al*., 2004). Note that *SIS2* was set aside to play the role as a latent factor when real data were analyzed. In particular, *SGS1* encodes a RecQ DNA helicase whose homologues in human cells include the *WRN*, *BLM* and *RECQ4* genes. Mutations in these genes lead to cancer-predisposition syndromes and symptoms resembling premature aging (Tong *et al*., 2001). Mutation of *SGS1* gene results in premature aging in yeast mother cells on the basis of a shortened life-span as well as genome instability. Hence, yeast and human share highly conserved molecular mechanisms in preventing aging process as well as maintaining genome integrity.

The steady state mRNA levels of the five genes (*SGS1, TOP1, MUS81*, *CSM3, SWE1* in Figure 3) in the corresponding null mutant strains (*sgs1Δ, top1Δ, mus81Δ, csm3Δ,* or *swe1Δ*) were compared to those in the wild type yeast strain. The results would allow one to examine if the cell could detect the loss of a gene and simply respond by increasing the expression level, for example steady state level of mRNA, of its compensatory genes. The steady state mRNA levels were determined by the quantitative real-time reverse transcription (RT)-polymerase chain reaction (PCR) method, which is more precise and reproducible than northern blot and DNA microarray. All five PCR primer pairs were validated with measurement of their mRNA present in the corresponding null mutants. The steady state levels of actin mRNA were used as the quantitative references between wild type and mutant strains.

Specifically, four replicates of steady state mRNA levels of a given gene were attained when its paralog gene or SSL partner is mutated (Mu) and a wild type (Wt), respectively. If following a gene's loss, its compensatory gene's expression decreases, we call this phenomenon transcriptional diminishment. Let μ_{Mu} and μ_{Wt} denote the mean mRNA levels of a given gene when its paralog gene or SSL partner was mutated and a wild type, respectively. To see if gene B is compensatory of or diminished by gene A, we test on whether μ_{Mu} of gene B is significantly larger or smaller than the associated μ_{Wt}, respectively. To test $H_0 : \mu_{Mu} = \mu_{Wt}$ versus $H_1 : \mu_{Mu} \neq \mu_{Wt}$, we conducted two-sample t-tests on all pairs of genes included in the RT-PCR experiments since we were only provided with the means and standard errors of the four replicates. For any t-test exceeding the 2-sided 95% critical values (thresholds), we declared the transcriptional compensation (diminishment) between the two genes significant, and drew a link between them accordingly in Figure 3.

The *sgs1Δ* null mutant is viable, indicating that yeast cell is capable of tolerating the loss of *SGS1* gene. This tolerance may rely on redundant genes or pathways that can compensate for the loss of one another. This phenomenon was referred previously as *transcriptional compensation*. While the existence of genetic compensation is well accepted, it is still not known how this phenomenon is achieved? In the case of *sgs1Δ* null mutant, not all compensatory genes can be conceived simply based upon the biochemical functions of their protein products. For example, the *CSM3* gene was originally identified from a genome-wide reverse genetic screen for accurate chromosome segregation during meiosis. Csm3 protein forms a ternary complex with Mrc1 and Tof1, which is required for efficient sister chromatid cohesion and DNA-repair checkpoint. Swe1 is a protein kinase that regulates the G2/M transition by inhibition of Cdc28 protein kinase activity. On the contrary, a few other compensatory genes are functionally related to Sgs1 proteins. The *TOP1* gene encodes DNA topoisomerase I (TopI) and the Mus81 protein functions as an endonuclease in complex with Mms4 protein. The Mus81-Mms4 complex, Sgs1-Top3-Rmi1 complex and the Srs2 DNA helicase likely involve in three alternative pathways to prevent formation of toxic recombination intermediates from single-stranded gaps created by DNA replication.

cDNA microarray data from the *alpha*, *cdc15* and *cdc28* experiments in Spellman *et al.* (1998) were applied to the four algorithms to infer the gene networks in Figure 3. The *Elu* data set was not included because it was synchronized differently from *alpha*, *cdc15* and cdc28. The experiment and control groups were mRNAs extracted from synchronized and non-synchronized yeast cultures, respectively. The synchronization was conducted by treating yeast cultures with alpha factor arrest and arrests of a temperature-sensitive mutant *cdc15* and mutant *cdc28*. A full description and complete data sets are available at http://cellcycle-www.stanford.edu. The red (R) and green (G) fluorescence intensities were measured from the mRNA abundance in the experiment group and control group, respectively. Log ratios of R to G were used to reconstruct the interaction networks. There are 18, 24 and 17 time points in *alpha*, *cdc15* and *cdc28* data sets with no replicates; these three datasets were aggregated to increase time points to 59.

Log ratios of the six genes' expression levels were fitted to SSEM with BIC, VBEM (Beal *et al*., 2005), MAPEM (Rangel *et al*., 2004) and LDS (Perrin *et al*., 2003) algorithms. Since not all possible pairs of (30) links were checked by RT-PCR experiments, the modified TPR is defined to be the percentage of the correctly predicted existing links from the confirmed existing links in Figure 3. The modified TPR of the top 1 model selected by SSEM with BIC is equal to 9/16 (56%). While the modified TPRs of VBEM and MAPEM are equal to 2/16 (13%) based on both 99% and 95% confidence intervals and 7/16 (44%), respectively; LDS is not able to confirm the existence of any links. The nine correctly predicted links of SSEM are *SIS2*⊣ *CSM3, SIS2* ⊣ *TOP1, SGS1*⊣*CSM3, SGS1* → *TOP1, CSM3*⊣*SGS1, CSM3*⊣*TOP1, MUS81*⊣ *CSM3, MUS81* → *TOP1* and *SWE1* → *TOP1*.

4 DISCUSSION

Among the six goodness-of-fit indices, BIC works best with SSEM. BIC assigns larger penalties to model complexity than AIC does, resulting in higher TNRs for all cases when inferring the simulated 6-gene network. These results may be due to the fact that the true network is relatively sparse; further simulation studies along these lines may clarify this issue. SSEM has the following advantages. SSEM can infer genetic networks using time course data with no replicates, as is shown in the implementation section. SSEM incor-

porates latent factors to reconstruct gene networks, which results in biologically realistic models. However, it does not assume any structure for latent factors; this reduces the total number of parameters to be estimated, hence it requires less data than state space models. However, SSEM shares one drawback with Bayesian networks. Both approaches assume that the vector of log ratios of gene expression $\mathbf{y}(t)$ follows a multivariate normal distribution. This assumption may limit its applications, although log ratios of gene expression do follow a normal distribution in most cases.

Thus far, Bayesian networks and structure equation modeling are the only two approaches that incorporate hidden variables into the genetic networks. In the implementation section, the reconstruction results of the 6-gene network using real microarray data show that SSEM performs better than three Bayesian network algorithms. However, the modified TPR for SSEM with BIC applied to the real data is about 56% which is lower than the 72.5% TPR when it was applied to simulated data under CNR=1.3 and 50 time points. The low modified TPR for SSEM applied to real data may be caused by several factors. First, the real data are nosier than the simulated data, especially as the former were aggregated from similar, but not identical, data sets. This aggregation was applied in Xie and Bentler (2003) and others, and it also resulted in some meaning gene networks in Xie and Bentler (2003) but the prediction accuracy could not be checked since no biological experiments was carried out. Second, the true regulations may not be linear. For example, it may take multiple genes to activate a target gene, and there may be saturation of the gene effects regulating other genes. Third, the gene-gene and latent factor-gene regulations may be captured by lag-k in time with $k > 1$ rather than lag-1. The latter two issues suggest further research that we intend to pursue in the future.

ACKNOWLEDGEMENTS

The authors thank Drs. Ting-Fang Wang and Chih-Hung Jen for constructive discussions in biology, especially Dr. Wang for providing us with the RT-PCR results. This work was supported in part by NSC grant 92-2118-M001-023 to G.S.S. and by thematic grant AS-TP 23-33 to G.S.S. and T.F.W.; C.Y.Y. was supported by NSC postdoctoral fellowship 92-2811-M001-037 and 93-2811-M001-071.

REFERENCES

Akaike, H. (1973). Information theory and an extension of the maximum likelihood principle. In Petrov, B. N. and Csaki, F. (eds.), 2nd International Symposium on Information Theory, Akademiai Kiado, Budapest, pp. 267-281.

Akutsua, T., Kuhara, S., Maruyamac, O. and Miyano, S. (2003). Identification of genetic networks by strategic gene disruptions and gene overexpressions under a Boolean model. *Theoretical Computer Science*, 298, 235-251.

Akutsu, T., Miyano, S. and Kuhara, S. (1999). Identification of genetic networks from a small number of gene expression patterns under the Boolean network model. *Pacific Symposium on Biocomputing*, 4, 17-28.

Ando, S., Sakamoto, E. and Iba, H. (2002). Evolutionary modeling and inference of gene network. *Information Sciences*, 145, 237-259.

Beal, M.J., Falciani, F., Ghahramani, Z., Rangel, C. and Wild, D.L. (2005). A Bayesian approach to reconstructing genetic regulatory networks with hidden factors. *Bioinformatics*, 21, 349-356.

Bentler, P. M. (1990) Comparative fit indices in structural equation models. *Psychological Bulletin*, 107, 238-246.

Cattell, R.B. (1966). The scree test for the number of factors. *Multivariate behavioural research*. 1, 245 - 276.

de la Fuente, A., Bing, N., Hoeschele, I. and Mendes, P. (2004). Discovery of meaningful associations in genomic data using partial correlation coefficients. *Bioinformatics*, 20, 3565-3574.

Friedman, N., Linial, M., Nachman, I. and Pe'er, D. (2000). Using Bayesian networks to analyze expression data. *Journal of Computational Biology*, 7, 601–620.

Guthke, R., Möller, U., Hoffmann, M., Thies, F. and Töpfer, S. (2005). Dynamic network reconstruction from gene expression data applied to immune response during bacterial infection. *Bioinformatics*, 21, 1626-1634.

Guttman, L. (1954). Some necessary conditions for common-factor analysis. *Psychometrika*, 19, 149-161.

Hartemink, A. J., Gifford, D. K., Jaakkola, T. S. and Young, R. A. (2002). Bayesian methods for elucidating genetic regulatory networks. *IEEE Intelligent Systems*, 17, 37–43.

Hu, L. T. and Bentler, P. M. (1995). Evaluating model fit. In Hoyle, R. H. (ed.) Structural Equation Modeling: Concepts, Issues, and Applications, Sage Publication: Thousand Oaks, California, USA, Ch. 5, pp. 76-99.

Jöreskog, K. G. (1969). A general approach to confirmatory maximum likelihood factor analysis. *Psychometrika*, *34*, 183-202.

Kaplan, D. (2000). Structural equation modeling: Foundation and extensions. Sage Publication: Thousand Oaks, California, USA.

Kikuchi, S., Tominaga, D., Arita, M., Takahashi, K. and Tomita, M. (2003). Dynamic modeling of genetic networks using genetic algorithm and S-system. *Bioinformatics*, 19, 643-650.

Kimura, S., Ide1, K., Kashihara, A., Kano, M., Hatakeyama, M., Masui, R., Nakagawa, N., Yokoyama, S., Kuramitsu, S. and Konagaya, A. (2005). Inference of S-system models of genetic networks using a cooperative coevolutionary algorithm. *Bioinformatics*, 21, 1154-1163.

Kline, R. B. (1998) Principles and practice of structural equation modeling. The Guilford Press: New York, NY, U.S.A.

Kyoda, K., Baba, K., Onami, S. and Kitano, H. (2004). DBRF–MEGN method: an algorithm for deducing minimum equivalent gene networks from large-scale gene expression profiles of gene deletion mutants. *Bioinformatics*, 20, 2662-2675.

Liang, S., Fuhrman, S. and Somogyi, R. (1998). REVEAL, a general reverse engineering algorithm for inference of genetic network architectures. *Pacific Symposium on Biocomputing*, 3, 18-29.

Muthén, L. K., & Muthén, B. O. (2004). Mplus User's Guide. Los Angeles, CA: Muthén & Muthén.

Perrin, B.E., Ralaivola, L., Mazurie, A., Bottani, S., Mallet, J. and d'Alché–Buc, F. (2003). Gene networks inference using dynamic Bayesian networks. *Bioinformatics*, 19, ii138-ii148.

Rangel, C., Angus, J., Ghahramani, Z., Lioumi, M., Sotheran, E., Gaiba, A., Wild, D.L. and Falciani, F. (2004) Modelling T-cell activation using gene expression profiling and state space models. *Bioinformatics*, 20, 1361–1372.

SAS Institute Inc. (1988). SAS/ETS user's guide. Cary, NC: Author.

Schäfer, J. and Strimmer, K. (2005). An empirical Bayes approach to inferring large-scale gene association networks. *Bioinformatics*, 21, 754-764.

Schwarz, G. (1978) Estimating the dimension of a model. *Annals of Statistics*, 6, 461-464.

Sclove, S. L. (1987). Application of model-selection criteria to some problems in multivariate analysis. *Psychometrika*, 52, 333-343

Shmulevich, I., Dougherty, E. R., Kim, S., and Zhang, W. (2002). Probabilistic Boolean networks: A rule-based uncertainty model for gene regulatory networks. *Bioinformatics*, 18, 261–274.

Shmulevich, I., Dougherty, E. R. and Zhang, W. (2002). Gene perturbation and intervention in probabilistic Boolean networks. *Bioinformatics*, 18, 1319–1331.

Tamada, Y., Kim, S. Y., Bannai, H., Imoto, S., Tashiro, K., Kuhara, S. and Miyano, S. (2003). Estimating gene networks from gene expression data by combining Bayesian network model with promoter element detection. *Bioinformatics*, 19, ii227-ii236.

Thomas, R., Mehrotra, S., Papoutsakis, E.T. and Hatzimanikatis, V. (2004). A model-based optimization framework for the inference on gene regulatory networks from DNA array data. *Bioinformatics*, 20, 3221-3235.

Tong, A. H. *et al.* (2001). Systematic genetic analysis with ordered arrays of Yeast deletion mutants. *Science*, 294, 2364-2366.

Tong, A. H. *et al.* (2004). Global mapping of the Yeast genetic interaction network. *Science*, 303, 808-813.

Tucker, L. R. and Lewic, C. (1973). A reliability coefficient for maximum likelihood factor analysis. *Psychometrika*, 38, 1-10.

Wagner, A. (2001). How to reconstruct a large genetic network from n gene perturbations in fewer than n^2 easy steps. *Bioinformatics*, 17, 1183-1197.

Wong, S. L. and Roth, F. P. (2005) Transcriptional compensation for gene loss. *Genetics*, published online 5 July, 2005; 10.1534/genetics.105.046060.

Wright, S. (1918). On the nature of size factors. *Genetics*, 3, 367-374.

Xie, J. and Bentler, P. M. (2003). Covariance structure models for gene expression microarray data. *Structural Equation Modeling. 10,* 566-582.

Zhou, X., Wang, X. and Dougherty, E. R. (2003). Construction of genomic networks using mutual-information clustering and reversible-jump Markov-chain-Monte-Carlo predictor design. *Signal Processing*, 83, 745–761

Zou, M. and Conzen, S.D. (2005). A new dynamic Bayesian network (DBN) approach for identifying gene regulatory networks from time course microarray data. *Bioinformatics*, 21, 71-79.

TABLES

Table 1. Performance of SSEM using various goodness-of-fit indices (GFI) applied to 6-gene networks generated under (*CNR, T*) = (2, 100). The "Top 1 model" ("Top 5 models") denote all top 1 (top 5) networks, based on the specified GFI value, in 100 experiments.

GFI	Top 1 model			Top 5 models		
	TPR (%)	TNR (%)	FPR (%)	TPR (%)	TNR (%)	FPR (%)
BIC	97.3	97.1	2.9	97.0	95.3	4.7
AIC	97.7	85.1	14.9	97.7	84.4	15.6
adjBIC	98.7	80.5	19.5	98.7	79.7	20.3
χ^2 -df	96.7	72.7	27.3	96.6	72.4	27.6
χ^2 /df	96.2	64.7	35.3	96.1	67.8	32.2
MSE	92.1	60.7	39.3	94.3	73.3	26.7

Table 2. Performance of SSEM using various goodness-of-fit indices (GFI) applied to 6-gene networks generated under (*CNR, T*) = (2, 50). The "Top 1 model" ("Top 5 models") denote all top 1 (top 5) networks, based on the specified GFI value, in 100 experiments.

GFI	Top 1 model			Top 5 models		
	TPR (%)	TNR (%)	FPR (%)	TPR (%)	TNR (%)	FPR (%)
BIC	84.6	87.7	12.3	83.9	86.2	12.9
AIC	88.7	78.9	21.1	88.0	78.0	21.1
adjBIC	89.7	60.6	39.4	88.5	62.5	36.7
χ^2 -df	89.3	66.7	33.3	89.5	67.0	33.0
χ^2 /df	90.2	61.4	38.6	88.5	64.1	35.1
MSE	69.4	69.7	30.3	76.5	71.5	27.0

Table 3. Performance of SSEM applied to 6-gene networks generated under *CNR*=1.3. The "Top 1 model" ("Top 5 models") denote the average result of all top-1 (top-5) networks, based on BIC or AIC scores, in 100 experiments.

		Top 1 model			Top 5 models		
		TP (%)	TN (%)	FP (%)	TP (%)	TN (%)	FP (%)
T=100/ GFI	BIC	84.9	90.0	10.0	84.5	88.6	11.4
	AIC	88.7	78.8	21.2	88.8	78.5	21.5
T=50/ GFI	BIC	73.5	83.5	16.5	72.5	82.4	16.0
	AIC	78.9	72.7	27.3	78.1	71.8	27.0

Table 4. Performance of SSEM with BIC applied to 10-gene networks generated under various (*CNR, T)* cases. The "Top 1 model" ("Top 5 models") denote the average result of all top-1 (top-5) networks based on the BIC score in 100 experiments.

(*CNR, T*)	Top 1 model			Top 5 models		
	TP (%)	TN (%)	FP (%)	TP (%)	TN (%)	FP (%)
(2, 100)	98.4	96.5	3.5	98.3	96.2	3.8
(2, 50)	85.7	89.6	10.4	85.8	89.2	10.8
(1.3, 100)	82.3	88.2	11.8	82.1	87.8	12.2
(1.3, 50)	72.9	87.4	12.6	74.1	87.3	12.7

FIGURE LEGENDS

Fig. 1. The diagram for linear dynamic factor model, where circles and squares denote latent factors and observed genes, respectively.

Fig. 2 The latent factor-gene and gene-gene regulations, where squares and circles denote genes and latent factors, respectively, and the short arrows pointing to the upper-right corners of $y_i(t)$ denote noises $\varepsilon_i(t)$, for $1 \le i \le n$.

Fig. 3 The 6-gene regulatory network, where $A \rightarrow B$ ($A \dashv B$) denotes `A diminishes (compensates) B ', and circles and squares denote latent factors and genes, respectively.

FIGURES

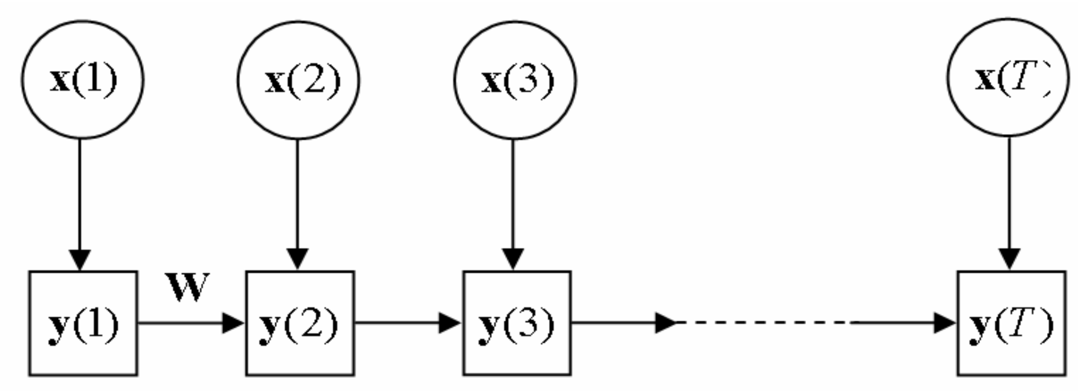

Figure 1.

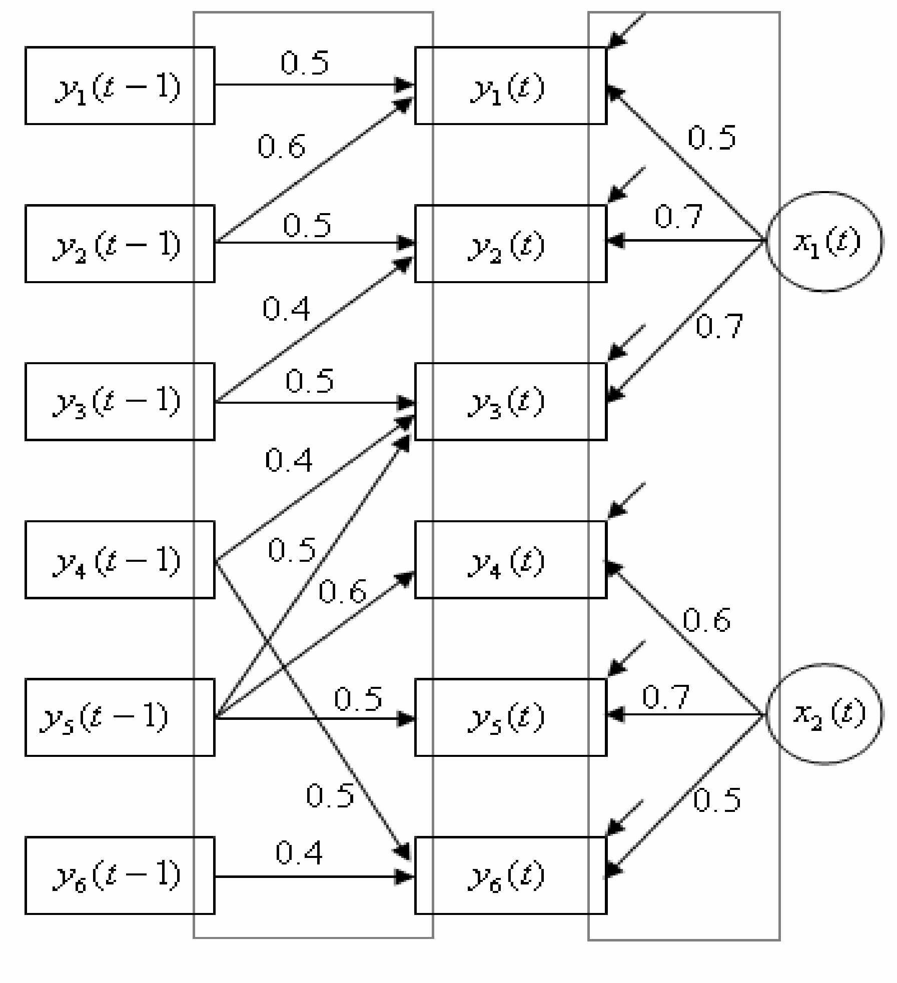
$y_1(t-1)$
$y_2(t-1)$
$y_3(t-1)$
$y_4(t-1)$
$y_5(t-1)$
$y_6(t-1)$
$y_1(t)$
$y_2(t)$
$y_3(t)$
$y_4(t)$
$y_5(t)$
$y_6(t)$
$x_1(t)$
$x_2(t)$
0.5
0.6
0.5
0.4
0.5
0.4
0.5
0.6
0.5
0.5
0.4
0.5
0.7
0.7
0.6
0.7
0.5

Figure 2.

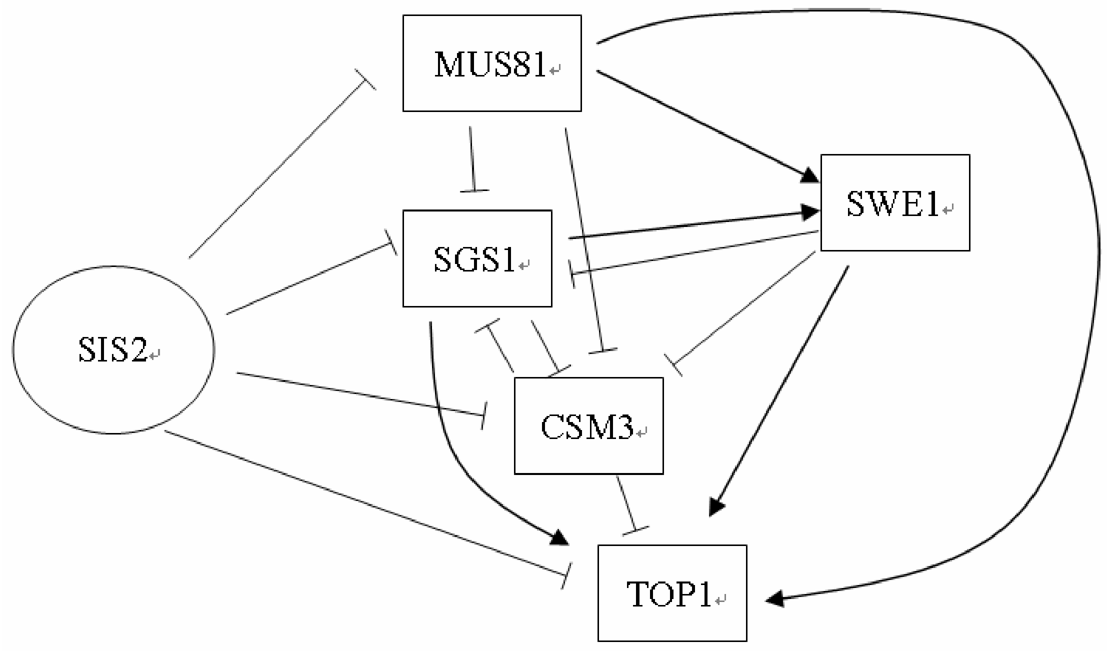

Figure 3.
