# Supplementary material for: Inferring transcriptional compensation interactions in yeast via stepwise structure equation modeling
Source: BMC Bioinformatics. 2008 Mar 3;9:134. doi: 10.1186/1471-2105-9-134 (PMC2323972; doi:10.1186/1471-2105-9-134)
Supplement: Additional file 2 — qRT-PCR. Description of the design of qRT-PCR experiments and how the experiments were conducted to confirm the predicted TC and TD interactions. [file 1471-2105-9-134-S2.pdf]

Supplementary file

Quantitative RT-polymerase chain reaction (qRT-PCR) experiments

qRT-PCR is a major development of PCR technology that enables reliable detection and measurement of products generated during each cycle of PCR process.

To check whether a gene pair has TC (or TD) interactions, we measured the qRT-PCR gene expression levels of gene B when its partner gene A was mutant and when gene A was wild-type (WT). By the definition of TC (TD), the expression levels of gene B should increase (decrease) when A was mutant versus when A was wild-type.

In order to verify the differences between experimental groups (knockout) and control group (WT) are significant or not, the aforementioned experiment was repeated 4 times for each group. Then a t-test was performed to check:

$$\begin{cases} H_0: \ \mu_C = \mu_E \\ H_1: \ \mu_C > \mu_E \ \text{ for testing TD} \end{cases}$$

$$(H_1: \ \mu_C < \mu_E \text{ for testing TC})$$

where μ_C and μ_E is the mean of gene expression in control group and experimental group, respectively, and $\alpha = 0.05$.

Due to the qRT-PCR results have not been published by our collaborator in biochemistry, we cannot release them.
