# Supplementary material for: Inferring transcriptional compensation interactions in yeast via stepwise structure equation modeling
Source: BMC Bioinformatics. 2008 Mar 3;9:134. doi: 10.1186/1471-2105-9-134 (PMC2323972; doi:10.1186/1471-2105-9-134)
Supplement: Additional file 3 — Simulation. The description of the 6- and 10-gene networks, and the results of EBVM applied to the two networks. [file 1471-2105-9-134-S3.pdf]

- **SSEM implementation** (pp.1-5)
- **VBEM in Beal et al. (2005) applied to data simulated from both 6- and 10-gene networks** (pp.5-11)

In this supplementary file, we first use simulated gene expression data to evaluate the performance of SSEM with various goodness-of-fit indices. Time course data from both a 6-gene and a 10-gene network are simulated. Next, SSEM is applied to a set of real time course microarray data to reconstruct a 6-gene network. Since the network topology, latent factors ($\mathbf{x}(t)$), gene-gene interactions ($\mathbf{W}$), and latent factor-gene regulations ($\mathbf{\Lambda}$) are well defined for the simulated data, exact quantitative performance can be accessed. The goal is to determine with which index among χ^2/df , χ^2-df , MSE, AIC, BIC, and adjBIC, SSEM performs best under various numbers of genes, sample sizes and noise levels. On the other hand, real microarray data contain true genetic interactions that may not be revealed in simulated networks. To see how SSEM performs relative to Bayesian networks, we apply SSEM and three Bayesian network algorithms from Beal *et al.* (2005), Rangel *et al.* (2004) and Perrin *et al.* (2003) to reconstruct a network that's regulations were confirmed by biological experiments. The final section consists of detail outputs of VBEM in Beal et al. (2005) applied to data simulated from both 6- and 10-gene networks.

Simulated time course data

Two genetic regulatory networks are simulated; a 6-gene network and a 10-gene network, and both have two latent factors. Experiences from the social sciences indicate that SEM works well provided that the ratio of observed variables to latent factors is at least 3, and our pilot studies also confirmed this. When the ratio of observed variables to latent factors is 5:2, the convergent solutions of the MLEs were only 58% for T = 50 and 81% for T = 100; but when the ratio is 6:2, the convergent solutions increased to 73% and 93% for T = 50 and 100, respectively. We adopted this 3 or higher ratios in the simulation studies. The linear dynamic factor model for the 6-gene network is defined as:

$$\begin{aligned}
y_1(t) &= 0.5x_1(t) + 0.5y_1(t-1) + 0.6y_2(t-1) + \varepsilon_1(t) \\
y_2(t) &= 0.7x_1(t) + 0.5y_2(t-1) + 0.4y_3(t-1) + \varepsilon_2(t) \\
y_3(t) &= 0.7x_1(t) + 0.5y_3(t-1) + 0.4y_4(t-1) + 0.5y_5(t-1) + \varepsilon_3(t) \\
y_4(t) &= 0.6x_2(t) + 0.6y_5(t-1) + \varepsilon_4(t) \\
y_5(t) &= 0.7x_2(t) + 0.5y_5(t-1) + \varepsilon_5(t) \\
y_6(t) &= 0.5x_2(t) + 0.5y_4(t-1) + 0.4y_6(t-1) + \varepsilon_6(t)
\end{aligned} \quad , (4)$$

where $x_1(t) \sim N(0,0.1)$, $x_2(t) \sim N(0,0.1)$, $y_i(0) \sim U(0,1)$, and $\varepsilon_i(t) \sim N(0,\sigma_i^2),\ i=1,\ldots,6$. Note that σ_i^2 is determined by the variance of $y_i(t)$ and a pre-specified noise level. The noise level is quantified by a contrast-to-noise ratio (CNR), defined as the ratio of the signal standard deviation to the noise standard deviation. *CNR* = 1.3 or 2.0 correspond to high or median noise levels, respectively. Similarly, the linear dynamic factor model for the 10-gene network is defined as follows.

$$\begin{aligned}
y_1(t) &= 0.7x_1(t) + 0.5y_1(t-1) + 0.45y_2(t-1) + \varepsilon_1(t) \\
y_2(t) &= 0.75x_1(t) + 0.5y_2(t-1) + 0.4y_3(t-1) + \varepsilon_2(t) \\
y_3(t) &= 0.7x_1(t) + 0.45y_3(t-1) + 0.5y_4(t-1) + \varepsilon_3(t) \\
y_4(t) &= 0.7x_1(t) + 0.45y_4(t-1) + 0.5y_5(t-1) + \varepsilon_4(t) \\
y_5(t) &= 0.85x_1(t) + 0.75y_5(t-1) + \varepsilon_5(t) \\
y_6(t) &= 0.7x_2(t) + 0.5y_6(t-1) + 0.45y_7(t-1) + \varepsilon_6(t) \\
y_7(t) &= 0.75x_2(t) + 0.5y_7(t-1) + 0.4y_8(t-1) + \varepsilon_7(t) \\
y_8(t) &= 0.7x_2(t) + 0.45y_8(t-1) + 0.5y_9(t-1) + \varepsilon_8(t) \\
y_9(t) &= 0.7x_2(t) + 0.45y_9(t-1) + 0.5y_{10}(t-1) + \varepsilon_9(t) \\
y_{10}(t) &= 0.85x_2(t) + 0.75y_{10}(t-1) + \varepsilon_{10}(t),
\end{aligned} \quad (5)$$

where $x_1(t) \sim N(0,0.1)$, $x_2(t) \sim N(0,0.1)$, $y_i(0) \sim U(0,1)$, and $\varepsilon_i(t) \sim N(0,\sigma_i^2),\ i=1,\ldots,10$. σ_i^2 is set by the variance of $y_i(t)$, and *CNR* = 1.3 or 2.0. As an example, Figure 3 illustrates the dynamics of the 6-gene network at time *t*, that is, the gene expressions at

time t are regulated by the latent factors at time t and the gene expressions at time $t-1$. Note that for each i, figures of the first 100 time points and the last 100 time points of $y_i(t)$ with T = 1000 show sinusoid patterns, and can be obtained from the authors upon request.

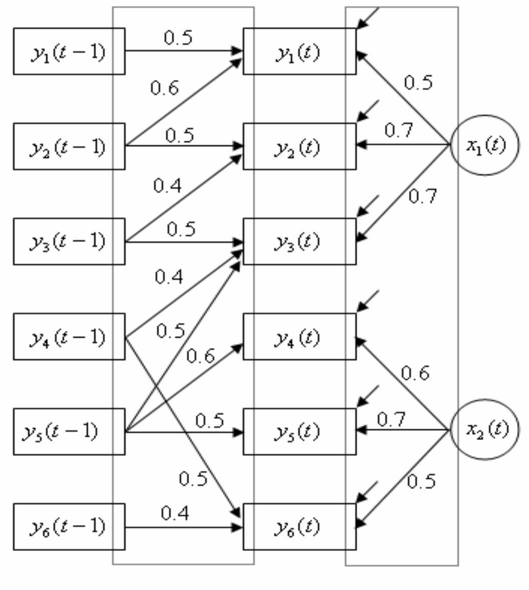

Fig. 3. The latent factor-gene and gene-gene regulations, where squares and circles denote genes and latent factors, respectively, and the short arrows pointing to the upper-right corners of $y_i(t)$ denote noises $\varepsilon_i(t)$, for $1 \le i \le n$.

Note that the networks in (4) and (5) are sparse which roughly follow the sparse property of *cis*-regulatory networks stated in van Someran *et al.* (2002). For each network in (4) and (5), time course data were simulated under four different conditions; sample sizes ($T = T_{\min}$, *50* or *100*) and noise levels (high or median), where T is the number of time points and $T_{\min} = 2n+1$. For the 6-gene network and a given set of (*CNR*, *T*), 100 experiments were carried out, and each simulation took about six days.

Tables 1 and 2 summarize the performance of SSEM with the six goodness-of-fit indices when (*CNR*, *T*) = (2, 100) and (2, 50), respectively. The averages of the true positive rate (TPR), true negative rate (TNR), and false positive rate (FPR) of all top 1 (top 5) networks, in terms of the goodness-of-fit index value, in 100 experiments are reported. TPR (also known as sensitivity) is the percentage of correctly predicted links from the total existing links (positives) in the simulated network. Likewise, TNR (specificity) is the percentage of correctly predicted non-existing links (negatives) out of the total non-existing links in the simulated network.

Taking 5% random error into account, SSEM with BIC clearly performs better than SSEM with the other five goodness-of-fit indices when (*CNR*, *T*) = (2, 100) and (2, 50). The TPRs and TNRs of SSEM with BIC are much higher than those of SSEM with adjBIC, MSE, χ^2-df or χ^2/df. TPRs and TNRs of SSEM with BIC range from 95% to 97% when (*CNR*, *T*) = (2, 100), and range from 84% to 88% when (*CNR*, *T*) = (2, 50). Both SSEM with χ^2-df and with χ^2/df tend to have high sensitivities (TPRs) but low specificities (TNRs) when (*CNR*, *T*) = (2, 100) and (2, 50). Similar observation may be made for SSEM with adjBIC when (*CNR*, *T*) = (2, 50). SSEM with MSE has the worst performance since its TPRs and TNRs ranging from 69% to 77% when (*CNR*, *T*) = (2, 50) though it performs similarly to SSEM with χ^2/df when (*CNR*, *T*) = (2, 100). Note that the TNR of `Top 1 model' resulted from SSEM with MSE is significantly lower than that of `Top 5 models' for data generated from the 6-gene network with *CNR* = 2. This shows that assigning no penalty on model complexity of MSE causes an over-fitting problem, namely, a network that has more links tends to have a better MSE score but it may not be closer to the true network.

Table 1. Performance of SSEM using various goodness-of-fit indices (GFI) applied to 6-gene networks generated under (*CNR*, *T*) = (2, 100). The "Top 1 model" ("Top 5 models") denote all top 1 (top 5) networks, based on the specified GFI value, in 100 experiments.

GFI	Top 1 model			Top 5 models		
	TPR (%)	TNR (%)	FPR (%)	TPR (%)	TNR (%)	FPR (%)
BIC	97.3	97.1	2.9	97.0	95.3	4.7
AIC	97.7	85.1	14.9	97.7	84.4	15.6
adjBIC	98.7	80.5	19.5	98.7	79.7	20.3
χ^2 -df	96.7	72.7	27.3	96.6	72.4	27.6
χ^2 /df	96.2	64.7	35.3	96.1	67.8	32.2
MSE	92.1	60.7	39.3	94.3	73.3	26.7

Table 2. Performance of SSEM using various goodness-of-fit indices (GFI) applied to 6-gene networks generated under (*CNR*, *T*) = (2, 50). The "Top 1 model" ("Top 5 models") denote all top 1 (top 5) networks, based on the specified GFI value, in 100 experiments.

GFI	Top 1 model			Top 5 models		
	TPR (%)	TNR (%)	FPR (%)	TPR (%)	TNR (%)	FPR (%)
BIC	84.6	87.7	12.3	83.9	86.2	12.9
AIC	88.7	78.9	21.1	88.0	78.0	21.1
adjBIC	89.7	60.6	39.4	88.5	62.5	36.7
χ^2 -df	89.3	66.7	33.3	89.5	67.0	33.0
χ^2 /df	90.2	61.4	38.6	88.5	64.1	35.1
MSE	69.4	69.7	30.3	76.5	71.5	27.0

Though their performances are close and they clearly outperform the other four, for all four cases, SSEM with BIC results in higher TNRs than SSEM with AIC, taking 5% random error into account. Due to the larger penalty on adding a link, BIC is more stringent than AIC in including a link in the network because BIC has significantly higher TNRs (namely lower FPRs). We further compare SSEM with BIC and SSEM with AIC under (*CNR*, *T*) = (1.3, 100) and (1.3, 50), respectively; the results are in Table 3. When CNR decreases from 2 to 1.3 (data are noiser), TPRs and TNRs of both SSEMs deteriorate about 10% for both sample sizes. However, BIC remains superior to AIC. Furthermore, even with a high noise level (*CNR* = 1.3), SSEM with BIC still results in TPRs and TNRs about 85% and 89%, respectively for *T* = 100, and about 73% and 82%, respectively for *T* = 50.

Table 3. Performance of SSEM applied to 6-gene networks generated under *CNR*=1.3. The "Top 1 model" ("Top 5 models") denote the average result of all top-1 (top-5) networks, based on BIC or AIC scores, in 100 experiments.

	Top 1 model	Top 5 models

		TP (%)	TN (%)	FP (%)	TP (%)	TN (%)	FP (%)
T=100/ GFI	BIC	84.9	90.0	10.0	84.5	88.6	11.4
	AIC	88.7	78.8	21.2	88.8	78.5	21.5
T=50/ GFI	BIC	73.5	83.5	16.5	72.5	82.4	16.0
	AIC	78.9	72.7	27.3	78.1	71.8	27.0

The simulation studies on the 6-gene network show that SSEM with BIC performs relatively well to very well under the four cases. To see how network complexity influences SSEM, we ran SSEM with BIC on data simulated from the 10-gene network in (5), with 50 experiments under the four cases of (*CNR*, *T*). The simulation results are summarized in Table 4. In general, SSEM with BIC performances on the 10-gene network are not significantly different from those on the 6-gene network for all cases. For the 10-gene network, TPRs (TNRs) of the Top 1 model resulted from SSEM with BIC are about 98%, 86%, 82%, and 73% (97%, 90%, 88% and 87%), for (*CNR*, *T*) = (2, 100), (2, 50), (1.3, 100), and (1.3, 50), respectively.

Table 4. Performance of SSEM with BIC applied to 10-gene networks generated under various (*CNR, T)* cases. The "Top 1 model" ("Top 5 models") denote the average result of all top-1 (top-5) networks based on the BIC score in 100 experiments.

(*CNR, T*)	Top 1 model			Top 5 models		
	TP (%)	TN (%)	FP (%)	TP (%)	TN (%)	FP (%)
(2, 100)	98.4	96.5	3.5	98.3	96.2	3.8
(2, 50)	85.7	89.6	10.4	85.8	89.2	10.8
(1.3, 100)	82.3	88.2	11.8	82.1	87.8	12.2
(1.3, 50)	72.9	87.4	12.6	74.1	87.3	12.7

To see how SSEM performs when time course is short, we further ran SSEM with BIC and AIC on those 6-gene and 10-gene networks with *T* = 13 and *T* = 21, respectively. Note that for a n-gene network, *T* = 2n+1 is the minimum number of time points required (denoted as $T_{\min}$) for proper estimation of $\hat{\Sigma}$ in (3) and hence for all parameters in the model. The performances are summarized in Table 5. For the 6-gene network, TPRs (TNRs) of the Top 1 model obtained by SSEM with BIC under *T* = 13 are about 81% and 77% (79% and 75%), for CNR=2 and 1.3, respectively. These results are equivalent to those obtained under *T* = 50. For the 10-gene network, TPRs (TNRs) of the Top 1 model resulted from SSEM with BIC are about 62% and 60% (67% and 62%) for *CNR* = 2 and 1.3, respectively; these TPRs and TNRs are lower than those obtained under *T* = 50 but they are all above 60%. For all cases under *T* = 13 and 21 except the case of *CNR* = 2, SSEM with AIC and SSEM with BIC have equivalent results. Note that in the case of *CNR* = 2, SSEM with AIC has about 9% higher TPRs and TNRs than SSEM with BIC, although this could be due to random error. These results are satisfactory. All simulation studies were conducted by PCs with Pentium IV 3.4 GHz and 2.5 GB RAM. Our algorithm is written in Visual C++ 6.0 and it calls SAS 8.2 and Mplus 3.0. For the 6-gene (10-gene) network, on average one case took about 10 (135) and 29 (235) minutes for *T* = 50 and *T* = $T_{\min}$, respectively.

Table 5. Performance of SSEM applied to 6-gene and 10-gene networks when sample size (T) is small.

(*n*, *CNR*, *T*)	GFI	Top 1 model TPR (%)	Top 1 model TNR (%)	Top 1 model FPR (%)	Top 5 models TPR (%)	Top 5 models TNR (%)	Top 5 models FPR (%)
(6, 2.0, 13)	BIC	80.9	79.1	20.9	71.8	63.5	36.5
	AIC	81.8	80.4	19.6	72.1	64.0	36.0
(6, 1.3, 13)	BIC	77.1	75.4	24.6	68.9	61.0	39.0
	AIC	78.0	76.7	23.3	69.5	61.5	38.5
(10, 2.0, 21)	BIC	62.0	67.0	33.0	59.6	66.5	33.5
	AIC	71.5	77.0	23.0	58.3	76.1	23.9
(10, 1.3, 21)	BIC	60.0	62.0	38.0	50.7	73.5	26.5
	AIC	64.0	62.0	38.0	52.0	72.5	27.5

Performance of VBEM (Beal *et al.*, 2005) applied to data simulated from Eq. (4) and Eq. (5)

6 genes generated with CNR 1.3

Gene-gene interaction (*D* matrix)

	Gene1	Gene2	Gene3	Gene4	Gene5	Gene6
Gene1	-0.2454	0.3479	0.1332	-0.1623	0.1045	0.0825
Gene2	-0.1091	0.2159	0.2389	0.0191	0.3154	-0.0939
Gene3	0.0455	0.1831	0.1685	-0.0411	0.3275	0.0276
Gene4	0.1191	0.0211	0.012	0.0026	0.1541	0.2082
Gene5	0.1523	0.0706	-0.0038	-0.2161	0.2161	-0.0876
Gene6	-0.1004	0.0313	0.0103	-0.0167	0.0722	0.0309

All predicted interactions are not significant.

Gene-gene interaction (*D* matrix, 95% level of significance)

	Gene1	Gene2	Gene3	Gene4	Gene5	Gene6
Gene1	0	0	0	0	0	0
Gene2	0	0	0	0	0	0
Gene3	0	0	0	0	0	0
Gene4	0	0	0	0	0	0
Gene5	0	0	0	0	0	0
Gene6	0	0	0	0	0	0

Latent factor – gene interaction

	CA*
Gene1	0.1739
Gene2	-0.1574
Gene3	-0.0988
Gene4	0.1897

Gene5	-0.1251
Gene6	0.3098

* CA represents latent factor.

The number of hidden states: 2

The number of correctly predicted links: 0

True-positive rate = 0/11 = 0% (checked against 11 synthetic links in the simulated data)

☨6 genes generated with CNR 2.0

Gene-gene interaction (*D* matrix)

	Gene1	Gene2	Gene3	Gene4	Gene5	Gene6
Gene1	0.0728	0.6556	0.1142	0.3231	0.0874	-0.0099
Gene2	0.1877	0.3499	0.3276	0.2753	0.2032	0.171
Gene3	-0.3512	-0.2893	0.1013	0.0163	0.0076	0.156
Gene4	-0.0592	-0.251	-0.0568	0.0826	0.1378	-0.0553
Gene5	0.0013	-0.1961	-0.0735	0.0709	0.0823	-0.1748
Gene6	0.0093	-0.31	0.1198	-0.0501	-0.001	-0.0507

Links marked in red color denotes significant links at 95% level of significance.

Gene-gene interaction (*D* matrix, 95% level of significance)

	Gene1	Gene2	Gene3	Gene4	Gene5	Gene6
Gene1	0	0	0	0	0	0
Gene2	0	0	0	0	0	0
Gene3	-0.3512	0	0	0	0	0
Gene4	0	0	0	0	0	0
Gene5	0	0	0	0	0	0
Gene6	0	0	0	0	0	0

The symbol "**o**" denotes correctly predicted links, and symbol "**x**" denotes incorrectly predicted links.

Latent factor – gene interaction

	CA*
Gene1	-0.6069
Gene2	0.0648
Gene3	0.7274
Gene4	-0.3457
Gene5	-0.5457
Gene6	0.269

* CA represents latent factor.

The number of hidden states: 2

The number of correctly predicted links: 0

True-positive rate = 0/11 = 0% (checked against 11 synthetic links in the simulated data)

†10 genes generated with CNR 1.3

Gene-gene interaction (*D* matrix)

	Gene1	Gene2	Gene3	Gene4	Gene5	Gene6	Gene7	Gene8	Gene9	Gene10
Gene1	0.0143	0.0989	0.123	-0.2047	0.0317	-0.1508	-0.0089	-0.1513	0.0345	0.0335
Gene2	0.0767	0.253	0.4784	-0.1292	0.2863	-0.2442	0.2143	-0.0838	0.1021	0.137
Gene3	-0.3103	-0.129	0.0125	0.0357	0.0277	0.0638	-0.0745	-0.2337	-0.0552	-0.037
Gene4	-0.1073	-0.4359	-0.2135	0.1335	-0.2262	0.0088	0.0337	-0.1057	-0.3075	-0.1742
Gene5	-0.1468	-0.0901	0.029	-0.24	-0.1268	-0.1084	0.1198	-0.2246	-0.273	-0.0745
Gene6	0.1194	0.1005	-0.0818	-0.716	-0.4771	-0.4637	0.2344	-0.0741	-0.4607	-0.0946
Gene7	-0.3245	-0.1096	-0.1661	-0.1621	-0.226	-0.1417	-0.2135	-0.2238	-0.0856	-0.0681
Gene8	-0.0423	-0.1837	0.2803	0.129	0.019	-0.531	0.0922	-0.0891	-0.0233	0.0104
Gene9	0.0888	-0.0647	0.3927	0.1046	0.1634	-0.329	0.2958	-0.0214	-0.0711	0.0396
Gene10	0.019	0.049	0.1354	0.003	0.1467	0.077	0.0771	-0.0602	0.0236	0.0305

Links marked in red color denotes significant links at 95% level of significance.

Gene-gene interaction (*D* matrix, 95% level of significance)

	Gene1	Gene2	Gene3	Gene4	Gene5	Gene6	Gene7	Gene8	Gene9	Gene10
Gene1	0	0	0	0	0	0	0	0	0	0
Gene2	0	0	0.4784 **o**	0	0	0	0	0	0	0.137
Gene3	0	0	0	0	0	0	0	0	0	0
Gene4	0	-0.4359	0	0	0	0	0	0	0	-0.1742
Gene5	0	0	0	0	0	0	0	0	0	0
Gene6	0	0	0	-0.716	-0.4771	0	0	0	-0.4607	0
Gene7	-0.3245	0	0	0	0	0	-0.2135 **x**	0	0	0
Gene8	0	0	0	0	0	-0.531	0	0	0	0
Gene9	0	0	0	0	0	0	0	0	0	0
Gene10	0	0	0	0	0	0	0	0	0	0

The symbol "**o**" denotes correctly predicted links, and symbol "**x**" denotes incorrectly predicted links.

Latent factor – gene interaction

	CA*
Gene1	0.4728
Gene2	0.1602
Gene3	-0.3199
Gene4	0.179
Gene5	-0.3456
Gene6	-0.4855
Gene7	-0.2576
Gene8	0.7312
Gene9	0.3437

Gene10	0.0774

* CA represents latent factor.

The number of hidden states: 10

The number of correctly predicted links: 1

True-positive rate = 1/18 = 6% (checked against 18 synthetic links in the simulated data)

†10 genes generated with CNR 2.0

Gene-gene interaction (*D* matrix)

	Gene1	Gene2	Gene3	Gene4	Gene5	Gene6	Gene7	Gene8	Gene9	Gene10
Gene1	0.3232	-0.041	0.2196	0.0975	0.2529	-0.0747	-0.2973	0.1189	-0.5067	-0.3069
Gene2	0.1233	-0.0812	0.0596	0.0084	0.2647	-0.0646	-0.2126	-0.0949	-0.5086	-0.4151
Gene3	-0.4313	-0.3083	-0.2212	-0.003	0.1815	0.0944	0.0535	-0.2571	0.2387	-0.3391
Gene4	-0.2673	-0.0489	0.2331	0.0865	0.1183	-0.0282	-0.0064	-0.2663	0.0382	-0.1264
Gene5	-0.2123	0.0209	0.1972	-0.0652	-0.1011	0.046	0.2242	-0.0687	0.0882	0.1353
Gene6	0.2722	0.2217	0.1611	-0.0026	-0.1055	-0.0763	-0.0384	0.1137	-0.2038	0.1973
Gene7	0.3488	-0.1184	-0.109	-0.0679	-0.1153	-0.1177	-0.2925	0.6142	-0.3431	0.1337
Gene8	0.2219	-0.1714	-0.1543	0.1888	0.0571	-0.0385	-0.3466	0.3989	0.1992	0.022
Gene9	0.0281	-0.0662	0.3757	0.2038	0.0031	-0.2724	-0.4611	0.2479	-0.181	0.1423
Gene10	0.1698	-0.0721	-0.1536	0.1677	0.2689	-0.0957	-0.3806	-0.0848	-0.1511	-0.3414

Links marked in red color denotes significant links at 95% level of significance.

Gene-gene interaction (*D* matrix, 95% level of significance)

	Gene1	Gene2	Gene3	Gene4	Gene5	Gene6	Gene7	Gene8	Gene9	Gene10
Gene1	0	0	0	0	0	0	0	0	0	0
Gene2	0	0	0	0	0	0	0	0	0	0
Gene3	-0.4313	-0.3083	0	0	0	0	0	0	0	0
Gene4	0	0	0	0	0	0	0	0	0	0
Gene5	0	0	0	0	0	0	0.2242	0	0	0
Gene6	0	0.2217	0	0	0	0	0	0	0	0
Gene7	0	0	0	0	0	0	0	0.6142 **o**	0	0
Gene8	0	0	0	0	0	0	0	0	0	0
Gene9	0	0	0	0	0	-0.2724	0	0	0	0
Gene10	0	0	0	0	0	0	0	0	0	0

The symbol "**o**" denotes correctly predicted links, and symbol "**x**" denotes incorrectly predicted links.

Latent factor – gene interaction

	CA*
Gene1	-0.9795
Gene2	-0.4813
Gene3	0.4649
Gene4	-0.0937
Gene5	-0.1766
Gene6	-0.2852
Gene7	-0.1768
Gene8	0.0429
Gene9	-0.1506

Gene10	0.177

* CA represents latent factor.

The number of hidden states: 11

The number of correctly predicted links: 1

True-positive rate = 1/18 = 6% (checked against 18 synthetic links in the simulated data)
