# Supplementary material for: Inferring transcriptional compensation interactions in yeast via stepwise structure equation modeling
Source: BMC Bioinformatics. 2008 Mar 3;9:134. doi: 10.1186/1471-2105-9-134 (PMC2323972; doi:10.1186/1471-2105-9-134)
Supplement: Additional file 4 — BayesianNW. The 6-gene network predicted by the three Bayesian network algorithms in Beal et al. (2005), Rangel et al. (2004) and Perrin et al. (2003). [file 1471-2105-9-134-S4.pdf]

- 6-genes network predicted by three Bayesian network algorithms in Beal *et al.* (2005), Rangel *et al.* (2004) and Perrin *et al.* (2003).

Figure of 6-gene regulatory network

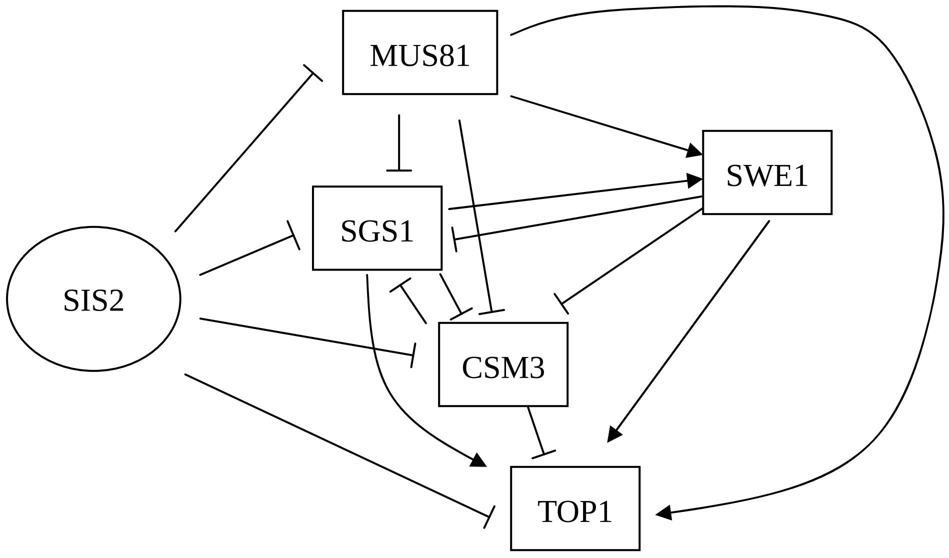

Figure 4. The 6-gene regulatory network, where $A \rightarrow B$ ($A \dashv B$) denotes `A and B are TD (TC)' .

Log ratios of expression levels of the 6 genes were fitted to VBEM (Beal *et al.*, 2005). Since data were integrated from alpha, cdc15 and cdc28 datasets, thus each gene has 59 time points with no replication. The parameters of VBEM were determined as follows:

Number of hidden state**:** 1; Number of iteration: 1000.

After VBEM converged, the interactions between the 6 genes can be predicted by the *CB*+*D* matrix. However, since the values in the *C* and *B* matrices are negligible, the final decisions of VBEM can be determined by the *D* matrix. The prediction results were subject to t-test with 95% and 99% level of significance, to distinguish significant interactions (links). The *D* matrix is summarized in the second table. The first column contains genes which are regulated by the genes in the first row of the matrix. Figure 4 shows 16 links (interactions) that were confirmed by qRT-PCR experiments. The predicted interactions, which are significant, are highlighted by red color. The prediction accuracies based on 95% and 99% level of significance are both equal to 2/16 (12.5%). Since not all possible pairs of links were checked by qRT-PCR experiments, the modified true positive rate (mTPR) is defined to be the percentage of the correctly predicted existing links from the confirmed existing links. The mTPR of VBEM is equal to 2/16 (12.5%).

†VBEM (Beal *et al.*, 2005)

Gene-gene interaction (*D* matrix)

	SWE1	CSM3	TOP1	MUS81	SGS1
SWE1	**0.3619**	0.0548	0.0832	-0.0451	**-0.4006**
CSM3	-0.0334	**0.3030**	**0.1329**	**-0.1845**	**-0.5024**
TOP1	0.0329	0.0294	**0.2081**	-0.0328	**-0.0630**
MUS81	**-0.0280**	-0.0241	**0.0390**	-0.0099	-0.0658
SGS1	**0.2946**	-0.0607	0.0025	0.0875	0.2117

The first column contains genes which are regulated by the genes in the first row of the matrix.
Significant links at 95% level of significance are highlighted in red color.

Gene-gene interaction (*D* matrix, 95% and 99% level of significance)

	SWE1	CSM3	TOP1	MUS81	SGS1
SWE1	0.3619	0	0	0	-0.4006 **x**
CSM3	0	0.3030	0.1329	-0.1845 **o**	-0.5024 **o**
TOP1	0	0	0.2081	0	-0.0630 **x**
MUS81	-0.0280	0	0.0390	0	-0.0658
SGS1	0.2946 **x**	0	0	0	0.2117

The symbol "**o**" denotes correctly predicted links, and symbol "**x**" denotes incorrectly predicted links.

Latent factor – gene interaction

	CA*
SWE1	-0.1311×10^{-41}
CSM3	-0.1804×10^{-41}
TOP1	-0.0365×10^{-41}
MUS81	-0.1089×10^{-41}
SGS1	0.0736×10^{-41}

* CA represents latent factor.

The number of correctly predicted links: 2

True-positive rate = 2/16 = 13% (checked against 16 qRT-PCR confirmed interactions)

†<u>MAPEM</u> (Rangel *et al.*, 2004)

Gene-gene interaction (*CB*+*D* matrix)

	SWE1	CSM3	TOP1	MUS81	SGS1
SWE1	0.8427	-0.0421	0.0257	-0.3071 **x**	-0.0095 **x**
CSM3	-0.0912 **o**	0.9756	0.0149	-0.1780 **o**	-0.0055 **o**
TOP1	-0.0496 **x**	-0.0133 **o**	1.0081	-0.0969 **x**	-0.0030 **x**
MUS81	-0.1134	-0.0304	0.0186	0.7786	-0.0069
SGS1	0.0517 **x**	0.0138 **x**	-0.0085	0.1009 **x**	1.0031

The symbol "**o**" denotes correctly predicted links, and symbol "**x**" denotes incorrectly predicted links.

Gene-gene interaction (*D* matrix)

	SWE1	CSM3	TOP1	MUS81	SGS1
SWE1	0.8656	-0.0358	-0.0454	-0.2077	0.0883
CSM3	-0.0779	0.9793	-0.0263	-0.1204	0.0512
TOP1	-0.0424	-0.0113	0.9857	-0.0655	0.0279
MUS81	-0.0969	-0.0258	-0.0327	0.8503	0.0637
SGS1	0.0442	0.0118	0.0149	0.0682	0.9710

Latent factor – gene interaction

	CA*
SWE1	-0.0368
CSM3	-0.0213 **o**
TOP1	-0.0116 **o**
MUS81	-0.0265 **o**
SGS1	0.0121 **x**

* CA represents latent factor.

The symbol "**o**" denotes correctly predicted links, and symbol "**x**" denotes incorrectly predicted links.

The number of correctly predicted links: 7

True-positive rate = (4+3)/16 = 44% (checked against 16 qRT-PCR confirmed interactions)

†LDS (Perrin *et al.*, 2003)

Gene-gene interaction (all are not significant)

	CA*	SWE1	CSM3	TOP1	MUS81	SGS1
CA	-0.0010	0.0016	-0.0013	0.0015	0.0009	0.0006
SWE1	0.0005	0.0026	0.0009	-0.0007	0.0021	-0.0034
CSM3	0.0001	-0.0008	0.0011	-0.0009	0.0029	0.0013
TOP1	-0.0007	-0.0018	-0.0019	0.0011	-0.0012	-0.0008
MUS81	-0.0000	0.0010	0.0016	0.0006	-0.0039	0.0003
SGS1	-0.0012	-0.0028	-0.0010	0.0011	-0.0013	0.0009

* CA represents latent factor.

The number of correctly predicted links: 0

True-positive rate = 0/16 = 0% (checked against 16 qRT-PCR confirmed interactions)
