# Supplementary material for: Inferring transcriptional compensation interactions in yeast via stepwise structure equation modeling
Source: BMC Bioinformatics. 2008 Mar 3;9:134. doi: 10.1186/1471-2105-9-134 (PMC2323972; doi:10.1186/1471-2105-9-134)
Supplement: Additional file 5 — Multi-AR(1). The result of fitting multivariate AR(1) straightforwardly to the real data for the 6-gene network. [file 1471-2105-9-134-S5.pdf]

REPORT

The multivariate time series first order auto-regressive model for the real data is

$$Y_{1,t} = \phi_{11}Y_{1,t-1} + \phi_{12}Y_{2,t-1} + \phi_{13}Y_{3,t-1} + \phi_{14}Y_{4,t-1} + \phi_{15}Y_{5,t-1} + \varepsilon_{1,t}$$
$$Y_{2,t} = \phi_{21}Y_{1,t-1} + \phi_{22}Y_{2,t-1} + \phi_{23}Y_{3,t-1} + \phi_{24}Y_{4,t-1} + \phi_{25}Y_{5,t-1} + \varepsilon_{2,t}$$
$$Y_{3,t} = \phi_{31}Y_{1,t-1} + \phi_{32}Y_{2,t-1} + \phi_{33}Y_{3,t-1} + \phi_{34}Y_{4,t-1} + \phi_{35}Y_{5,t-1} + \varepsilon_{3,t}$$
$$Y_{4,t} = \phi_{41}Y_{1,t-1} + \phi_{42}Y_{2,t-1} + \phi_{43}Y_{3,t-1} + \phi_{44}Y_{4,t-1} + \phi_{45}Y_{5,t-1} + \varepsilon_{4,t}$$
$$Y_{5,t} = \phi_{51}Y_{1,t-1} + \phi_{52}Y_{2,t-1} + \phi_{53}Y_{3,t-1} + \phi_{54}Y_{4,t-1} + \phi_{55}Y_{5,t-1} + \varepsilon_{5,t}$$

In matrix notation, the model can be expressed as

$$\mathbf{Y}_t = \mathbf{\Phi}\mathbf{Y}_{t-1} + \mathbf{E}_t$$

where, $\mathbf{\Phi}$ is a 5x5 coefficients matrix, and $\mathbf{E}_t$ is a 5×1 vector of white noises for the five stationary series, assuming mean is zero and serially uncorrelated. However, at each time point t, the components of $\mathbf{E}_t$ can be correlated.

$\mathbf{E}_t \sim (\mathbf{0}, \mathbf{\Sigma}_E)$, assuming $E(\mathbf{E}_t) = \mathbf{0}$ and $Cov(\mathbf{E}_t) = \Sigma_E$.

The total number of parameters of the model includes 25 (= 5×5) parameters in the $\mathbf{\Phi}$ matrix. Then the AR(1) model is fitted with the statistical software R by solving the system of linear difference (Yule-Walker) equations.

In R, multivariate AR is fitted:

```
# import data
SSEM <- read.table(file="F:/SSEM/alpha.txt",header=TRUE)

# centering
for(i in 1:ncol(SSEM)){ SSEM[,i] <- SSEM[,i] - mean(SSEM[,i]) }

# create a time series object
SSEM.ts <- ts(data=SSEM, start=1, end=11, frequency=1)

# fit a multivariate AR1 model
SSEM.ar <- ar(SSEM.ts, aic=FALSE, order.max=1)
```

Stationarity of series was checked by the plots of autocorrelation. For all 5 series of interest, the auto-correlation function has the form of a mixture of “quick decay” and “damped sinusoid”, which is the typical behavior of a stationary series. The sinusoid patterns in ACF plots suggest that AR(2) may be fitted for some genes. However, this sample size (only 11 time points) is too small to fit a AR(2) model.

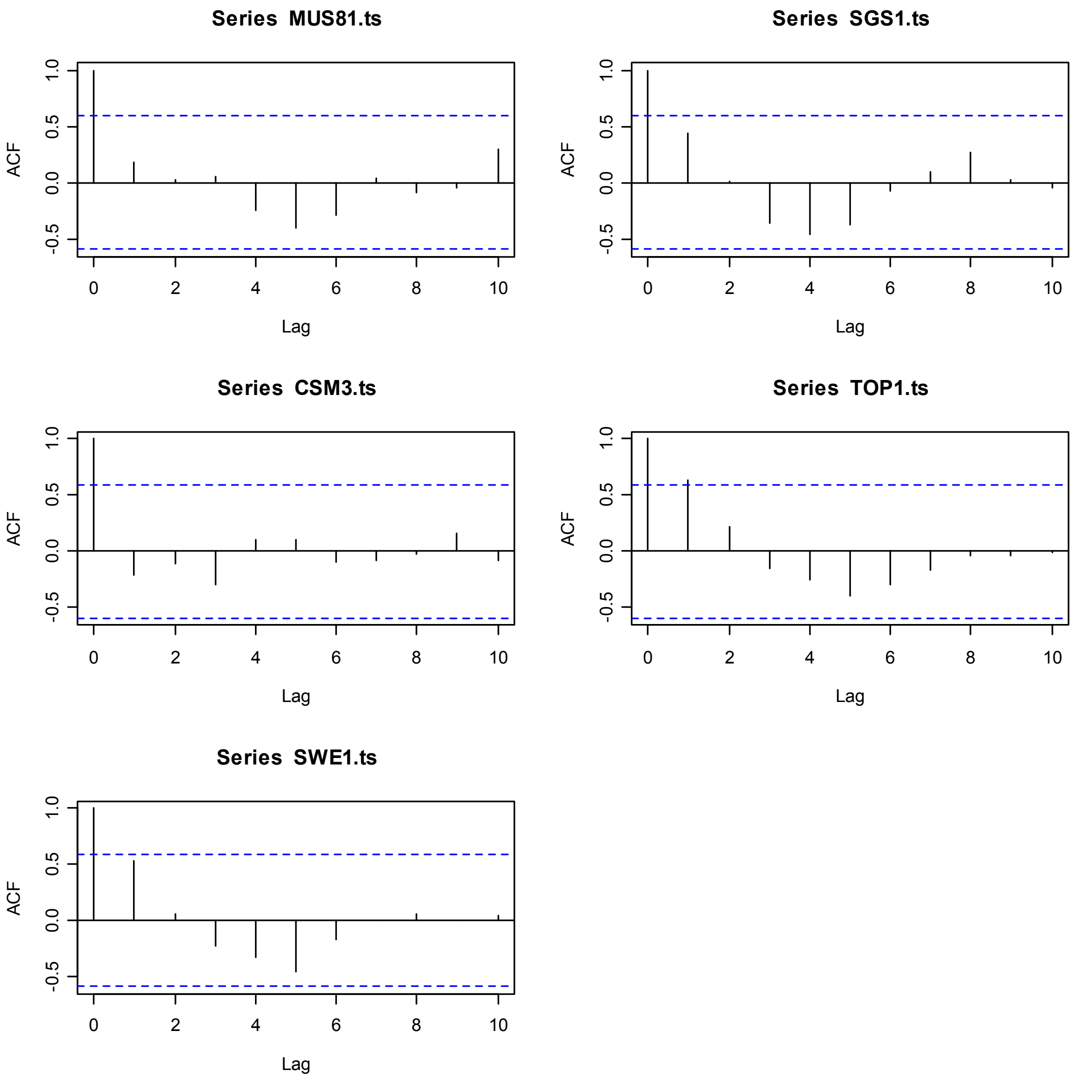

The residuals from the 5 short series show no strong pattern, and centered around zero. Some points of residual deviated from the center zero, but all of them are within the 95% interval based on the estimates of variance-covariance matrix $\mathbf{\Sigma}_E$.

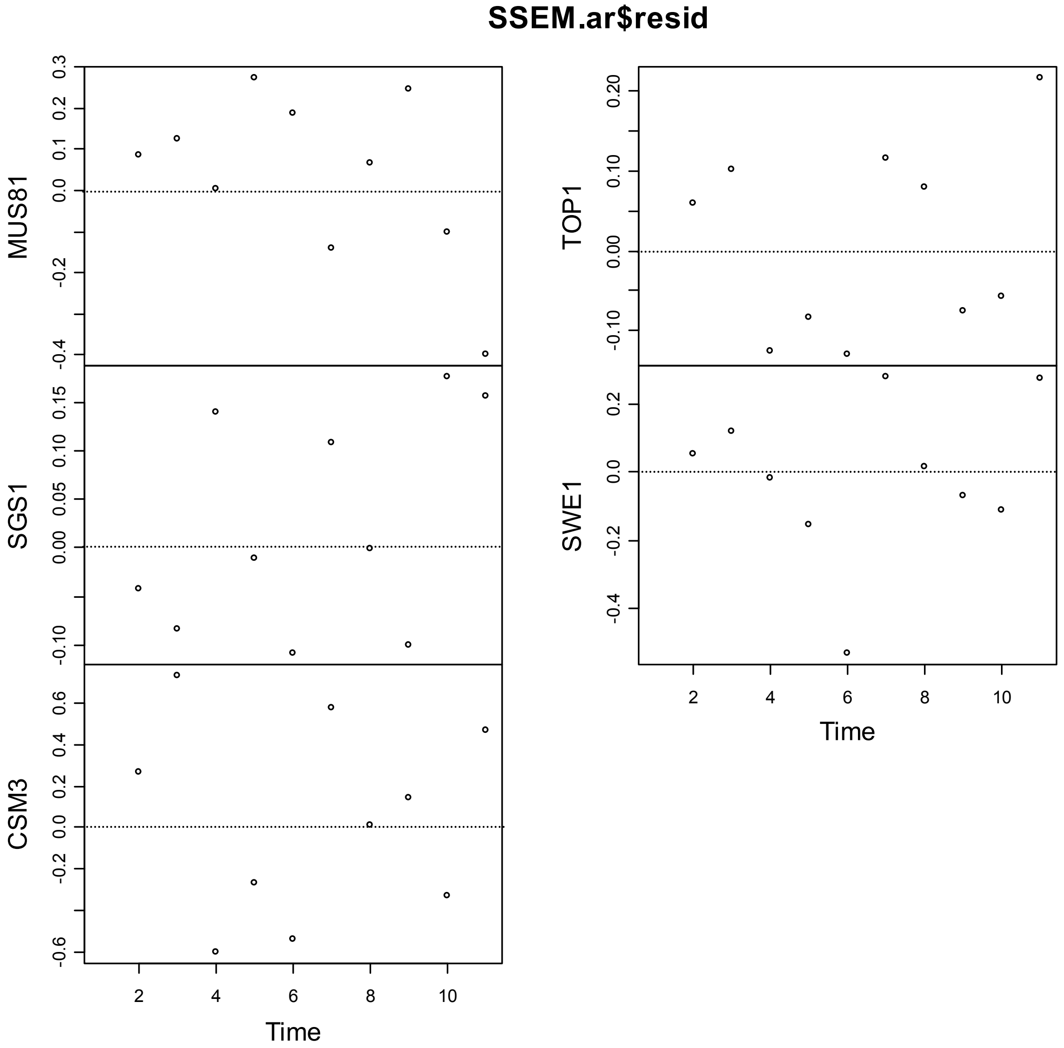

Estimate of variance-covariance matrix Σ_E

	MUS81	SGS1	CSM3	TOP1	SWE1
MUS81	0.61338	-0.05392	-0.04109	-0.11893	-0.45448
SGS1	-0.05392	0.16776	0.07303	0.04294	0.03097
CSM3	-0.04109	0.07303	2.22796	0.46134	0.59527
TOP1	-0.11893	0.04294	0.46134	0.13418	0.19362
SWE1	-0.45448	0.03097	0.59527	0.19362	0.64671

Parameters in the coefficient $\mathbf{\Phi}$ matrix were estimated and significance of $\hat{\phi}_{ij}$ is determined by the corresponding z-test. Let $\boldsymbol{\varphi}$ be the vector consisting all the columns of the $\mathbf{\Phi}$ matrix in order, and T be the number of time points in observations.

Then, $\sqrt{T}(\hat{\boldsymbol{\varphi}} - \boldsymbol{\varphi})$ has a multivariate normal distribution asymptotically,

namely $\sqrt{T}(\hat{\boldsymbol{\varphi}} - \boldsymbol{\varphi}) \rightarrow N(\mathbf{0},\ \mathbf{\Gamma}_y^{-1} \otimes \mathbf{\Sigma}_{\mathrm{E}})$, where $\mathbf{\Gamma}_y = \mathrm{var}(\mathbf{y}_t) = \sum_{j=0}^{\infty} \boldsymbol{\varphi}^j \mathbf{\Sigma}_{\mathrm{E}} (\boldsymbol{\varphi}^j)'$,

$\otimes$ denotes the Kronecker product of the matrices, and T is the number of time points

(T=11). Since there is no exact result, we use asymptotic variance of

$\hat{\boldsymbol{\varphi}} = (\mathbf{\Gamma}_y^{-1} \otimes \mathbf{\Sigma}_{\mathrm{E}})/\mathrm{T}$, which was estimated by $(\hat{\mathbf{\Gamma}}_y^{-1} \otimes \hat{\mathbf{\Sigma}}_{\mathrm{E}})/\mathrm{T}$, where $\mathbf{\Gamma}_y$ was

approximated by $\hat{\mathbf{\Gamma}}_y = \sum_{j=0}^{k} \hat{\boldsymbol{\varphi}}^j \hat{\mathbf{\Sigma}}_{\mathrm{E}} (\hat{\boldsymbol{\varphi}}^j)'$, and k=10. Those parameters ($\hat{\phi}_{ij}$) which are significant and have the same sign as the true links are regarded as true positives.

Predicted $\mathbf{\Phi}$ matrix:

	MUS81	SGS1	CSM3	TOP1	SWE1
MUS81	-0.25943	-0.10764	-0.43568	0.10571	0.07122
SGS1	-0.5560	-0.4183	-0.6831	0.3571	0.2848
CSM3	-0.02749	0.21089	-0.12781	-0.12127	-0.03085
TOP1	-0.1297	-1.7065	-0.6270	**1.6233**	1.5454
SWE1	-0.4187	0.7561	0.2351	-0.6017	-0.1322

* Red color denotes significant link at size α=0.05 by z-test

True links:

	MUS81	SGS1	CSM3	TOP1	SWE1
MUS81	NA	-	-	+	+
SGS1	NA	NA	-	+	+
CSM3	NA	-	NA	-	NA
TOP1	NA	NA	NA	NA	NA
SWE1	NA	-	-	+	NA

The estimated parameter does not match with any known link in the table above. Therefore, the true positive rate (TPR) of this multivariate AR(1) model equals to

TPR = 0/12 = 0

The P-value of $\hat{\phi}_{ij}$ is attached in the following table.

P-value of $\hat{\phi}_{ij}$:

		CoeffVec	AsymVar	z_statistic	P_value
1	$\hat{\phi}_{11}$	-0.25942862	1214.680540	0.0074436656	0.497
2	$\hat{\phi}_{12}$	-0.55596538	332.222046	0.0305023659	0.487
3	$\hat{\phi}_{13}$	-0.02749136	4412.077076	0.0004138802	0.499
4	$\hat{\phi}_{14}$	-0.12973995	265.715685	0.0079591215	0.496
5	$\hat{\phi}_{15}$	-0.41867905	1280.694691	0.0116992608	0.495
6	$\hat{\phi}_{21}$	-0.10763693	364.972516	0.0056341864	0.497
7	$\hat{\phi}_{22}$	-0.41827306	99.822062	0.0418645688	0.483
8	$\hat{\phi}_{23}$	0.21088613	1325.687551	0.0057919849	0.497
9	$\hat{\phi}_{24}$	-1.70648912	79.839035	0.1909835167	0.424
10	$\hat{\phi}_{25}$	0.75608481	384.807649	0.0385432795	0.484
11	$\hat{\phi}_{31}$	-0.43567666	358.467671	0.0230112015	0.49
12	$\hat{\phi}_{32}$	-0.68313839	98.042950	0.0689922811	0.472
13	$\hat{\phi}_{33}$	-0.12781191	1302.060042	0.0035420592	0.498
14	$\hat{\phi}_{34}$	-0.62696269	78.416077	0.0708009597	0.471

15	$\hat{\phi}_{35}$	0.23512036	377.949286	0.0120940979	0.495
16	$\hat{\phi}_{41}$	0.10571083	3.809419	0.0541614301	0.478
17	$\hat{\phi}_{42}$	0.35710024	1.041898	0.3498465376	0.363
18	$\hat{\phi}_{43}$	-0.12127435	13.836931	0.0326023618	0.486
19	$\hat{\phi}_{44}$	1.62329425	0.833324	1.7782397701	0.0376
20	$\hat{\phi}_{45}$	-0.60169399	4.016449	0.3002303101	0.382
21	$\hat{\phi}_{51}$	0.07122439	14.042900	0.0190064195	0.492
22	$\hat{\phi}_{52}$	0.28482836	3.840813	0.1453354757	0.442
23	$\hat{\phi}_{53}$	-0.03084699	51.007944	0.0043191066	0.498
24	$\hat{\phi}_{54}$	1.54542377	3.071934	0.8817421985	0.188
25	$\hat{\phi}_{55}$	-0.13218924	14.806089	0.0343538903	0.486
