# Supplementary material for: Inferring transcriptional compensation interactions in yeast via stepwise structure equation modeling
Source: BMC Bioinformatics. 2008 Mar 3;9:134. doi: 10.1186/1471-2105-9-134 (PMC2323972; doi:10.1186/1471-2105-9-134)
Supplement: Additional file 6 — SSL-TCNW. TC networks of SSL gene pairs. Description of how TC and TD interactions of SGS1 and RAD27 SSL gene pairs were predicted by SSEM. [file 1471-2105-9-134-S6.pdf]

The following figure is a genetic interaction network that represents the synthetic lethal/sick interactions determined by SGA analysis (Tong et al., 2001). Here genes are represented as nodes, and interactions are represented as edges that connect the nodes; 291 interactions and 204 genes are shown. Among these eight groups, inferring whether there is any TC or TD interactions of the SSL pairs involving the 51 genes in the lower two groups, linked to SGS1 and RAD27, and if yes, TC or TD interactions are of interest.

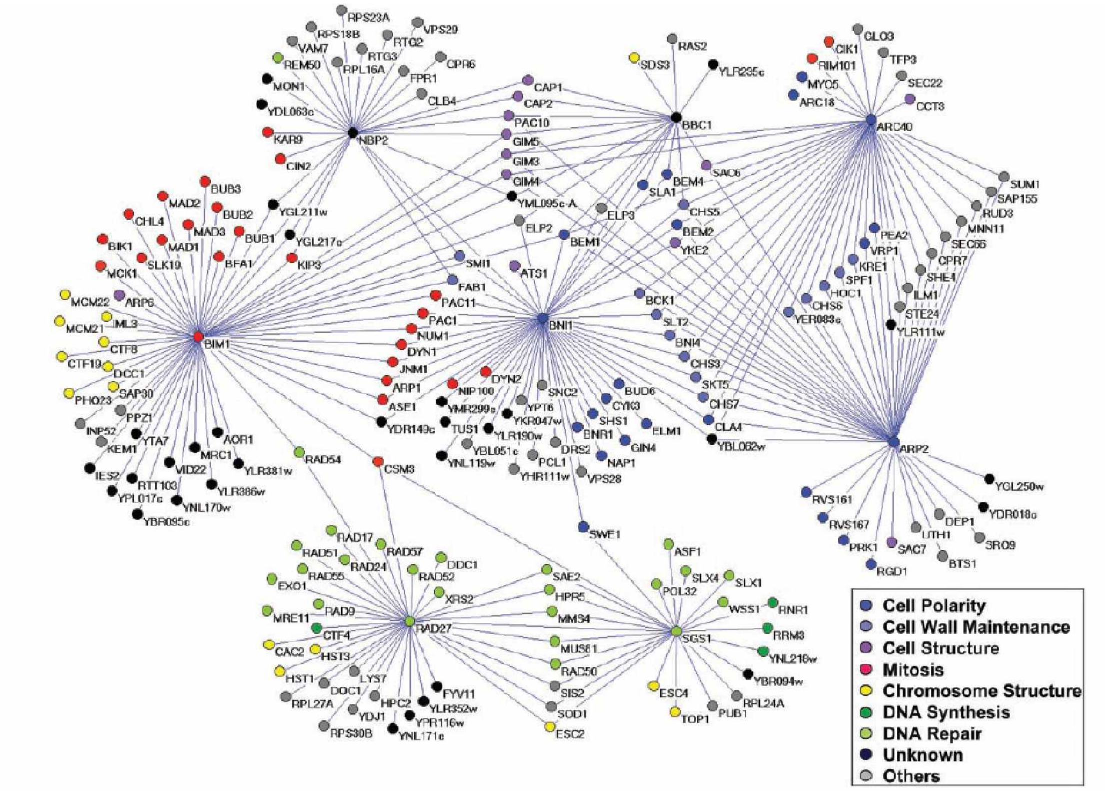

Fig. 3 of Tong *et al* . (2001).

The predictions for TC interactions from SSL pairs involving SGS1

Let G2-G22, G23 and G24 denote PLO32, ASF1, SLX4, …. RAD27 and TOP1 (clockwise in the following SGS1-SSL network). Since TC/TD interactions only exist in these SSL pairs, we set the influences Wj for non-SSL gene pairs to zero, and let W2-W24 be the influence of G2-G24 on SGS1 as follows.

(A1) SGS1(t)= $\sum_{k=1}^{K}\lambda_k F_k(t)+\sum_{i=2}^{24}W_i G_i(t-1)+\varepsilon(t).$

Among these 23 SSL gene pairs, we have q-RT-PCR results on 14 pairs only. SSEM predicts that SGS1 has TC interactions with ASF1, SLX4, WSS1, ESC4, SIS2,

RAD50, MUS81, MMS4, HPR5, SAE2 and SWE1, while interactions of SGS1 with POL32, SLX1, RNR1, YNL218W, RPL24A, PUB1, ESC4, ESC2, SOD1, MUS81 and CSM3 were predicted to be TD. Those marked by v (by x) were predicted correctly (incorrectly) in the network.

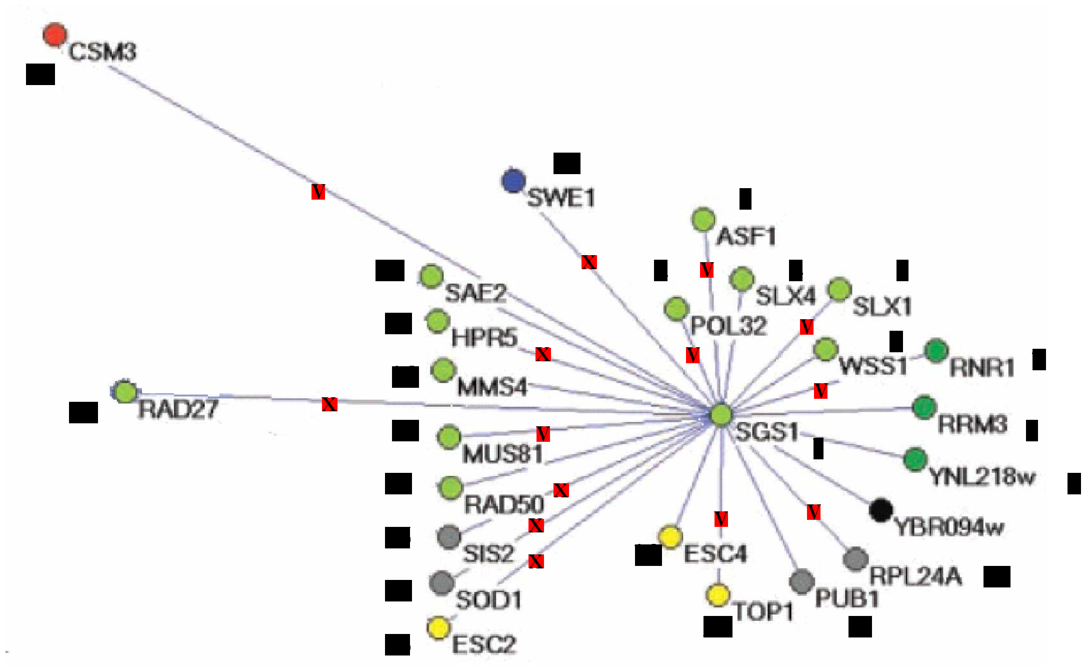

Figure of SGS1 SSL network.

Thus the modified TPR= 9/14. Note that the qRT-PCR experiments were done by measuring SGS1's expression level when G_i: Mutant or when G_i: wildtype, so we formulated Eq. (A1) that way.

Several predictions for TC interactions of SGS1 and its partners coincide with several known pathways of Sgs1

In the following, Sgs1, SGS1 and *sgs1* denote protein, wild-type and mutated gene of SGS1, respectively. Several functions of Sgs1 have been identified by *in vitro* biochemical tests or *in vivo* drug treatments and genetic screening in *sgs1* mutant cells constructed by gene mutation or disruption. Sgs1 plays important roles in yeast DNA repair, replication and S-phase checkpoint arrest. The role of Sgs1 in replication is thought to underlie the synthetic lethality/sickness of *sgs1* with *srs2*, *rrm3*, *slx1*, *slx4*, *mus81*, and *mms4* mutants (e.g. Fabre et al., 2002; Fricke and Brill, 2003). Checked against qRT-PCR results conducted by our collaborator, prediction of SSEM successfully uncovered TC and TD interactions of SGS1 with a few genes involved in DNA replication (e.g., SRS2, PLO32, RNR1, SLX1, MUS81 and TOP1), DNA repair

(e.g., RAD51 and RAD52), checkpoint arrest (e.g., RAD9) and chromosome segregation (e.g., CSM3). Among these interactions, all but SGS1-TOP1 are TC. These genetic interactions are consistent with the following experimental results from published literature.

Sgs1 and Srs2 are known redundant pathways in replication (Ira et al., 2003; Lee et al., 1999), for instance, Srs2 and Sgs1-Top3 suppress crossovers during double stand break repair in yeast. Further, defects in RAD51 and other homologous recombination genes suppressed synthetic lethality/sickness of the double mutant *sgs1Δ srs2Δ*. Slx1-Slx4 was found to be a second structure specific endonuclease functionally redundant with Sgs1/Top3 in Fricke and Brill (2003). It was reported in Frabe et al. (2002) that Sgs1/Top3 and Mus81/Mms4 complex were involved in both double-strand break repair and homologous recombination. This indicates that Sgs1/Top3 and Mus81/Mms4 are alternative pathways to resolve recombination intermediates. Onoda et al. (2001) found that Sgs1 participated in a RAD52-dependent recombinational pathway, while SGS1 and RAD52 were predicted to be TC by SSEM. Double mutant of *rad9* and *sgs1* showed some defects in maintaining genome stability (Schmidt and Kolodner, 2006) and homologous recombination (Schmidt et al., 2006). RAD9 and SGS1 were predicted and confirmed to be TC, which coincides with the result in Ooi et al. (2003) that Rad9 and Sgs1 interact genetically. Cells lacking Sgs1 frequently arrest as large-budded cells with a single nucleus in the mother cell, or "stuck" between mother and daughter cells which resulted in missegregation during mitosis (McVey et al., 2001; Lo et al., 2006), whereas Csm3 is required for DNA replication checkpoint and accurate chromosome segregation.

The predictions for interactions of RAD27 and its partners

Similarly, we inferred TC or TD interactions among SSL gene pairs of RAD27 as follows.

(A2) $$\text{RAD27(t)}= \sum_{k=1}^{K}\lambda_k F_k(t)+\sum_{2}^{15} W_i G_i(t-1)+\varepsilon(t)$$

where G_i, i=1,..,15 are the genes that are SSL to RAD27. The following genes HPR5, SGS1, MUS81, ESC2, HST1, HST3 and CSM3 are predicted to have TC interactions with RAD27, whereas RAD52, HPR5, SIS2, SOD2, HPC2, LYS7, RAD9, RAD51 and RAD54 are predicted to have TD interactions with RAD27.

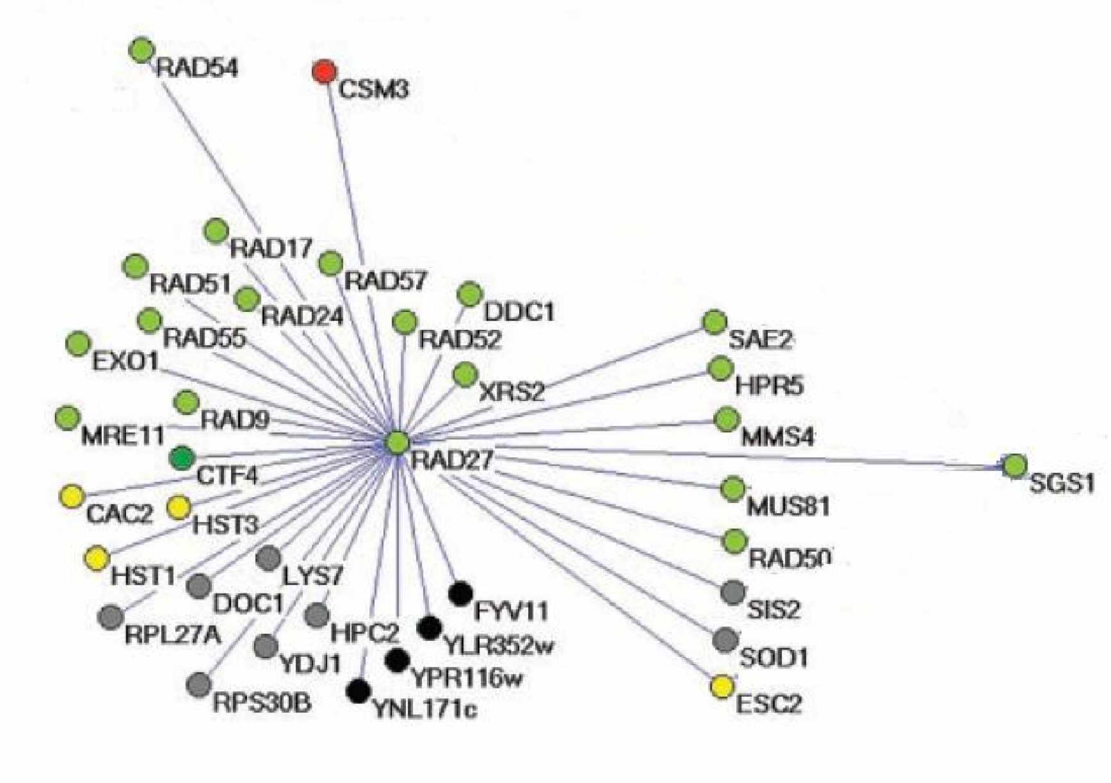

References:

Fabre, F., Chan, A., Heyer, W. D. and Gangloff , S. (2002). Alternate pathways involving Sgs1/Top3, Mus81/Mms4, and Srs2 prevent formation of toxic recombination intermediates from single-stranded gaps created by DNA replication. *Proc. Natl. Acad. Sci. USA*, 99, 16887-16892.

Fricke, W. M., and Brill, S. J. (2003). Slx1-Slx4 is a second structure-specific endonuclease functionally redundant with Sgs1-Top3. *Genes Dev.*, 17, 1768-1778.

Ira, G., Malkova, A., Liberi, G., Foiani, M. and Haber, J. E. (2003). Srs2 and Sgs1-Top3 suppress crossovers during double-strand break repair in yeast. *Cell,* 115, 401-411.

Lee, S. K., Johnson, R. E., Yu, S. L., Prakash, L. and Prakash, S. (1999). Requirement of yeast SGS1 and SRS2 genes for replication and transcription. *Science,* 286, 2339-2342.

Lo, Y. C., Paffett, K. S., Amit, O., Clikeman, J. A., Sterk, R., Brenneman, M. A. and Nickoloff, J. A. (2006). Sgs1 regulates gene conversion tract lengths and crossovers independently of its helicase activity *Mol. Cell. Biol.,* 26, 4086-4094.

McVey, M., Kaeberlein, M., Tissenbaum, H. A. and Guarente, L. (2001). The short life span of *Saccharomyces cerevisiae sgs1* and *srs2* mutants is a composite of normal aging processes and mitotic arrest due to defective recombination. *Genetics,* 157, 1531-1542.

Onoda, F., Seki, M., Miyajima, A. and Enomoto, T. (2001). Involvement of SGS1 in DNA damage-induced heteroallelic recombination that requires RAD52 in *Saccharomyces cerevisiae*. *Mol Gen Genet.*, 264, 702-708.

Ooi, S. L., Shoemaker, D. D. and Boeke, J. D. (2003). DNA helicase gene interaction network defined using synthetic lethality analyzed by microarray. *Nat Genet.*, 35, 277-286.

Schmidt, K. H. and Kolodner, R. D. (2006). Suppression of spontaneous genome rearrangements in yeast DNA helicase mutants. *Proc. Natl. Acad. Sci. USA*, 103, 18196-18201.

Schmidt, K. H., Wu, J. and Kolodner, R. D. (2006). Control of translocations between highly diverged genes by sgs1, the *Saccharomyces cerevisiae* homolog of the bloom's syndrome protein. *Mol. Cell. Biol.*, 26, 5406-5420.
