# Supplementary material for: Inferring transcriptional compensation interactions in yeast via stepwise structure equation modeling
Source: BMC Bioinformatics. 2008 Mar 3;9:134. doi: 10.1186/1471-2105-9-134 (PMC2323972; doi:10.1186/1471-2105-9-134)
Supplement: Additional file 7 — SSEM-algorithm. The zipped file consists of the standalone executable (.exe) file of SSEM. [file 1471-2105-9-134-S7.zip › SSEM-algorithm/simuall/fdr.plg]

```
# Build Log


### --------------------Configuration: fdr - Win32 Debug--------------------


### Command Lines

Creating temporary file "C:\DOCUME~1\JUILIN~1\LOCALS~1\Temp\RSP6D.tmp" with contents
[
kernel32.lib user32.lib gdi32.lib winspool.lib comdlg32.lib advapi32.lib shell32.lib ole32.lib oleaut32.lib uuid.lib odbc32.lib odbccp32.lib  kernel32.lib user32.lib gdi32.lib winspool.lib comdlg32.lib advapi32.lib shell32.lib ole32.lib oleaut32.lib uuid.lib odbc32.lib odbccp32.lib /nologo /subsystem:console /incremental:yes /pdb:"Debug/fdr.pdb" /debug /machine:I386 /out:"Debug/fdr.exe" /pdbtype:sept 
.\Debug\fdrProcessor.obj
.\Debug\fdr.obj
.\Debug\util.obj
]
Creating command line "link.exe @C:\DOCUME~1\JUILIN~1\LOCALS~1\Temp\RSP6D.tmp"

### Output Window

Linking...
LINK : fatal error LNK1104: cannot open file ".\Debug\fdrProcessor.obj"
Error executing link.exe.

### Results

fdr.exe - 1 error(s), 0 warning(s)
```
