# Supplementary material for: Inferring transcriptional compensation interactions in yeast via stepwise structure equation modeling
Source: BMC Bioinformatics. 2008 Mar 3;9:134. doi: 10.1186/1471-2105-9-134 (PMC2323972; doi:10.1186/1471-2105-9-134)
Supplement: Additional file 7 — SSEM-algorithm. The zipped file consists of the standalone executable (.exe) file of SSEM. [file 1471-2105-9-134-S7.zip › SSEM-algorithm/simuall/readme.doc]

**Note:**

1. SAS and m-plus packages are necessary for running SSEM as the main program, which has been coded in C-language, would be called by SAS and m-plus to generate data.

2. SAS version 8.2 and m-plus version 3.0 are recommended for smooth running.

**Steps:**

1. Please make sure that the software SAS and m-plus are installed and are running on path “C:\”

2. Please unzip the “src.zip”.

3. The file “sas.lnk” unzipped from the “src.zip” would automatically link with “sas.exe” on your PC.

**Data Input format:**

1. Please make sure that your data file has extension “.dat”, e.g. (example.dat)

2. Please make sure that data file has been formatted by “tab separate columns” and has been arrange as shown below:

G1

G2

G1

G2

Gk

Gk

T0

T1

:

:

Tn-1

T1

T2

:

:

Tn

T0

T1

:

:

Tn-1

T1

T2

:

:

Tn

T0

T1

:

:

Tn-1

T0

T1

:

:

Tn

…

…

3. Please make sure that the data file is in the folder called “data” which is following the path C:\SSEM-algorithm\simuall\data

**Output:**

1. Output result would be generated under C:\simuall\save_output\

2. True model is located in C:\SSEM-algorithm\simuall\true_models\

**Note:**

1. Configuration file named as “factor.cfg” is in C:\SSEM-algorithm\simuall\factor\ and consist of different important parameters which has been defined below:

- **Leadoff “#”** means that the line is comment.
- **MAX_ITERATION_NUM** is the parameter to set the number of iteration in the program.
- **NUM_REPLICATE** is the number of replicate in the data.
- **NUM_SUBJ** are the number of time point.
- **EST_SE_LOWER_BD** and **EST_SE_UPPER_BD** are the initial value for (a, b).
- **EST_SE_CUT_1** and **EST_SE_CUT_2** are two separate upper bounds.
- **TRUE_MDL_NVAR** is number of gene.
- **TRUE_MDL_NAME** and **TRUE_EQ_NAME** are true models.
- For different number of genes, users should make different files.
- **TRUE_MDL_NAME** is the model for true network and **TRUE_EQ_NAME** is its weight matrix.
- **EST_SE_ADJ_1**, **EST_SE_ADJ_2** and **EST_SE_ADJ_3** are the parameters to set the parameter **c**.
